# Exodus! Large-scale displacement and social adjustments of resident Atlantic spotted dolphins (*Stenella frontalis*) in the Bahamas

**DOI:** 10.1371/journal.pone.0180304

**Published:** 2017-08-09

**Authors:** Denise L. Herzing, Bethany N. Augliere, Cindy R. Elliser, Michelle L. Green, Adam A. Pack

**Affiliations:** 1 Wild Dolphin Project, Jupiter, Florida, United States of America; 2 Department of Biological Sciences, Florida Atlantic University, Boca Raton, Florida, United States of America; 3 Pacific Mammal Research, Anacortes, Washington, United States of America; 4 Department of Animal Science and Illinois Natural History Survey, University of Illinois at Urbana-Champaign, Urbana, Illinois, United States of America; 5 Departments of Psychology and Biology, University of Hawaii at Hilo, Hilo, Hawaii, United States of America; 6 The Dolphin Institute, Hilo, Hawaii, United States of America; University of Minnesota, UNITED STATES

## Abstract

Over the last 20 years, significant habitat shifts have been documented in some populations of cetaceans. On Little Bahama Bank (LBB) there are sympatric communities of resident Atlantic spotted dolphins (*Stenella frontalis*) and bottlenose dolphins (*Tursiops truncatus*), monitored since 1985. The size and social structure (three clusters: *Northern*, *Central*, *Southern*) have been stable among the spotted dolphin community with little immigration/emigration, even after large demographic losses (36%) following two major hurricanes in 2004. In 2013 an unprecedented exodus of over 50% (52 individuals) of the spotted dolphin community was documented. The entire *Central* cluster and a few *Northern* and *Southern* individuals relocated 161 km south to Great Bahama Bank (GBB), also home to two sympatric resident communities of spotted dolphins and bottlenose dolphins. During the late summer of 2013 and the summers of 2014 and 2015 both sites were regularly monitored but no former LBB dolphins returned to LBB. Uncharacteristic matriline splits were observed. Social analyses revealed random associations for those spotted dolphins and very little integration between spotted dolphins that moved to GBB (MGBB) and those dolphin resident to GBB (RGBB). Male alliances among spotted dolphins were present, with some altered patterns. On LBB, the operational sex ratio (OSR) was reduced (.40 to .25). OSR for MGBB and RGBB dolphins were similar (.45 and .43). A significant steady decrease in sea surface temperature and chlorophyll a (a proxy for plankton production) occurred on LBB leading up to this exodus. Similar trends were not present over the same period on GBB. The sudden large-scale shift of spotted dolphins from LBB to GBB in association with the gradual decline in certain environmental factors suggests that a possible “tipping point” was reached in prey availability. This study provides a unique view into social and genetic implications of large-scale displacement of stable dolphin communities.

## Introduction

Emigration and immigration patterns can greatly influence the origin and structure of social groups [1–2] as well as effect changes to previously stable groups, *e*.*g*. [3]. Very little is known about the effects of large-scale emigration events in social animals involving stable social clusters of individuals, where researchers have knowledge and long-term tracking of both the population from which immigrants arise and the population into which they join. Usually immigration events are at the individual, or small group level, where the association choices made by resident individuals can strongly affect the acceptance of immigrants into the population [1,4], ultimately affecting the grouping patterns and social structure [3]. Research on immigration events, whether large- or small-scale can be challenging due to the logistics involved in following individuals [5] and the fact that even for well-studied species immigration events are often rare [4]. This is particularly true for many cetacean species in which individuals typically spend most of their time underwater making them often difficult to track, and where some individuals (usually males) from distinct communities may pay occasional short-term visits to adjacent communities for purposes of mating and not immigration. Here, we describe the large scale and relatively rapid emigration of more than 50% of the community of long-term resident Atlantic spotted dolphins (*Stenella frontalis*) from Little Bahama Bank (LBB) to Great Bahama Bank (GBB) where another community of Atlantic spotted dolphins is resident.

The Wild Dolphin Project (WDP) has a unique long-term dataset with over 30 years of data for two sympatric communities of Atlantic spotted dolphins (*Stenella frontalis*) and bottlenose dolphins (*Tursiops truncatus*) on the northwestern edge of LBB. Data on Atlantic spotted dolphins includes life history and reproduction [6], correlations with sound and behavior [7–9], ultrasonic vocalizations [10], long-term community and social structure [11–12] nocturnal foraging [13], changes in social structure [14] and genetics [15–16]. The social structure of the resident community of bottlenose dolphins has also been documented over the decades [3,17–18].

Since 1991, annual immigration rates into the community of Atlantic spotted dolphins on LBB were low, with new births accounting for the majority of increases in the population [11]. The community was comprised of roughly 100 individuals in any given year prior to 2004, when two hurricanes struck the study area. Thirty six percent of the community was lost after these strong (category two and three) hurricanes [14] and these individuals have not been re-sighted to date. Following the hurricanes, approximately 67 community members remained. The population increased steadily thereafter and by late 2012 had nearly recovered to 85 individuals. Immigration after the hurricanes remained low (2005–2007: 2 noncalf individuals per year), consistent with pre-hurricane immigration (pre-hurricane 2002–2004: 2.3 noncalf individuals) [14]. This remained consistent in the following years (see results), indicating that the increase back to near normal numbers by 2012 was mainly due to births, not immigration into the population.

Following demographic upheaval after hurricane disturbance in 2004, differing social structure changes also occurred on LBB between the two closely related sympatric species of resident Atlantic spotted dolphins and bottlenose dolphins [3,14]. The long-term interspecies interactions of these two stable communities of dolphins have been documented [19–21], along with changes following the hurricanes [22].

Over the decades, and through the demographic upheaval of the two hurricanes mentioned earlier, three distinct and stable clusters (*Northern*, *Central*, *and Southern*) have been documented in the spotted dolphin community on LBB through genetics [23], social association data [11–12,14], and GIS distribution and home range analysis [24]. From 1985–2012 (28 years) both the communities of sympatric spotted and bottlenose dolphins on LBB have been resident to the area, showing strong site fidelity.

Data have also been collected in both winter (1998 through the winter of 2004) and summer months, on the resident communities of Atlantic spotted and bottlenose dolphins on GBB located approximately 50 km south of LBB. During this time, no photo identification matches were made between LBB and GBB spotted dolphins. Although consistent yearly data is unavailable for GBB, photographic evidence of site fidelity exists (see results).

Sometime between Sept 12, 2012 and May 28, 2013 (between summer field seasons), a major shift in the distribution of spotted dolphins occurred on LBB. The *Central* cluster of LBB spotted dolphins (45 dolphins) and 5 individuals from the *Northern* and 2 from the *Southern* clusters were found on GBB, and have remained there through the 2015 field season. In theory, this unprecedented shift can have large implications on the social and genetic structure of the larger population.

The large scale emigration/immigration that occurred between the two dolphin communities in this study provides a unique view into the social and genetic implications of a long-term stable social cluster from one community moving into an established community on another sandbank, and how the original community fairs after their exodus (with no subsequent immigration into that community). This paper discusses the preliminary observations, the social and genetic implications of this unprecedented emigration on these two sympatric communities, and the possible factors that may have contributed to the large-scale movement.

## Materials and methods

### Study areas

#### Little Bahama Bank (LBB)

**Fig 1:**
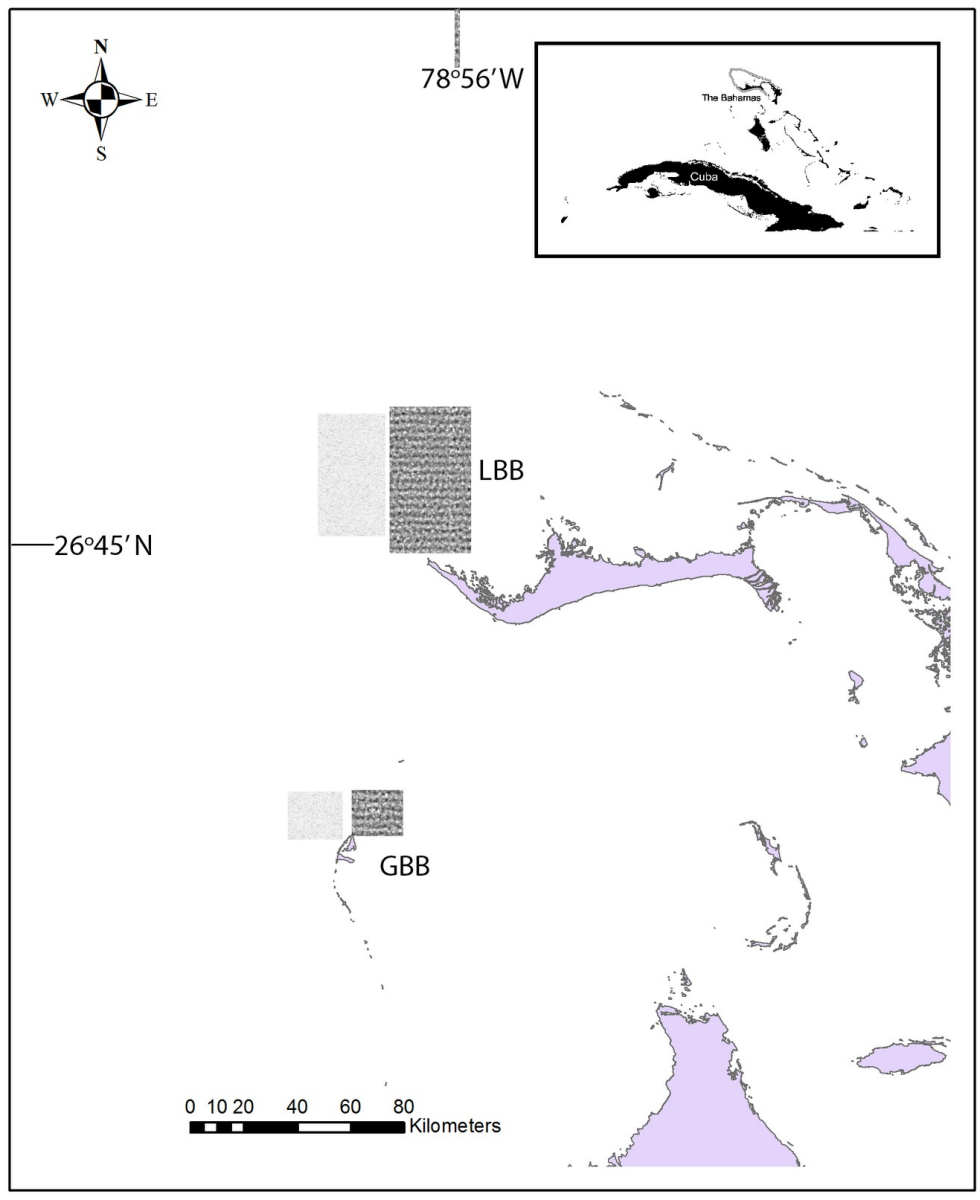
Map of the study area in the northern Bahamas. Little Bahama Bank (LBB) and Great Bahama Bank (GBB) in the northern Bahamas. Land masses (Grand Bahama Island–LBB) and Bimini–GBB) are in solid colors and are south of shaded areas. Oceanographic data was collected from dark shaded boxes that represent areas on the sandbank (shallow water), and lightly shaded boxes represent areas off the sandbank (adjacent deep water) for both LBB and GBB.

LBB is north of Grand Bahama Island and 64 km from the east coast of Florida (Fig 1). The sandbank is shallow, between 6–16 m deep and surrounded by deep water (steep drop off to over 500m into the Gulf Stream), and encompasses 480km^2^ (spanning 60 km north to south and 8km east to west).

#### Great Bahama Bank (GBB)

GBB is south of LBB from the southern end of Grand Bahama Island across deep water approximately 50 km to the beginning of the northern sandbank of GBB. This sandbank is larger than LBB, and encompassed twice the search area, 960km^2^ (Fig 1). The western edge of this sandbank is similar to LBB, with depths between 6-16m deep with deep water and a steep drop off to over 500m into the Gulf Stream on the western edge.

### Data collection—Effort

Data have been collected on the community of dolphins on LBB since 1985; the dolphins are habituated to the presence of boats and people in the water. Data were collected from May to September each year (average 80–100 d/yr) in all but rough weather conditions (over Beaufort 3 and/or intense rain squalls) from 0700 to 2000 in shifts of one person/one hour or 2 persons/two hours. Observers scanned an arc of 180° while underway and 360° while anchored. Past field effort on LBB (including number of days at sea, encounters, days with encounters, total hours and percent of time underway/anchored) for 1991–2002 can be found in [11], and for 2002–2007 in [14].

Data have also been collected (following the same protocol stated above for LBB) on the community of dolphins on GBB prior to the exodus (summer of 1998: 13 encounters, and the winters of 1999: 13 encounters, 2000: 18 encounters, 2001: 13 encounters, 2002: 4 encounters, and 2003: 13 encounters). Adjusted effort between LBB and GBB occurred since the summer of 2013 when over 50% of the long-term resident spotted dolphins on LBB were noted as missing from LBB. After surveys on LBB and the adjacent GBB, it was determined that these previously resident LBB individuals (primarily the *Central* cluster) moved to GBB. Survey time was split between these two sandbanks for the years of 2013–2015 summer field seasons. Table 1 shows the effort and time spent on both LBB and GBB over the recent three year summer field season periods.

**Table 1:** Effort for LBB and GBB 2013–2015.

Year	LBB	LBB Total #encounters	LBB Total duration	GBB	GBB Total #encounters	GBB total duration
	# days			# days		
2013	18	19	1158	15	14	1033
2014	12	23	1360	24	44	2328
2015	13	19	1152	11	21	939
Totals	43	61	3670	50	79	4300

LBB–Little Bahama Bank, GBB–Great Bahama Bank. Duration is given in minutes.

### Data collection—Individual, group, and community identification

Atlantic spotted dolphins have four developmental color phases described by [25] for the pantropical spotted dolphin (*Stenella attenuata*) and adapted for Atlantic spotted dolphins by [6]. The four age classes include: two-tones (calves, ≤4 yr), speckled (juveniles, 4–9 yr), mottled (young adult, 10–16 yr) and fused (adult, ≥16 yr). Every individual was assigned to an age class and these data were updated each year. Individual identification was accomplished using spotting patterns along with additional marks such as nicks and scars on the dorsal fin, flukes, pectoral fins and marks or scars on the body. Males were sexed by a gap between the genital slit and the anus, or observation of an erection. Females were sexed by observation of mammary slits or observation of nursing by a calf. Sex was determined for 98.5% of the LBB community, and 83.6% for GBB, and verified multiple times for all individuals seen more than once.

A group was defined as all dolphins in sight, moving in the same direction and typically involved in the same activity [26]. An encounter was defined as a group of dolphin observable underwater for more than 2–3 min [11]. The final group size/identified individuals were a product of in-water identification and photo-identification afterwards. Encounter methods including group size and individual ID followed the same methods described in [11–12].

For basic group analysis Atlantic spotted dolphins were categorized according not only to their location (LBB or GBB) but according to their *Community ID* as follows: LBB dolphins found on LBB, LBB dolphins found on GBB, GBB dolphins on GBB, LBB/GBB dolphins together on GBB. Bottlenose dolphins were categorized as single species in two locations (BN on LBB, BN on GBB) or as a bottlenose/spotted mixed group in the two locations (Mixed on LBB, Mixed on GBB).

For social analyses individual spotted dolphins were labeled as those resident/remaining on LBB after the displacement (RLBB), those resident to GBB (RGBB), and those that moved from LBB to GBB (MGBB).

This research was done under a permit issued by the Department of Fisheries in Nassau Bahamas. All research was observational and no samples were collected or experimental manipulation occurred during this study.

### Social structure analysis

Coefficients of association (CoAs) were calculated using the half-weight index [27] with the software program SOCPROG 2.5 [28]. Data were pooled into 3 year groupings: 1. 2007–2009 (a follow up after the before/after hurricanes analyses [14] to determine whether any further changes occurred, or remained stable), 2. 2010–2012 (three years prior to the displacement event and 3. 2013–2015 (3 years after the displacement event). An individual spotted dolphin was placed in the age class category that they were in for the majority of the pooled time frame (*i*.*e*. two or more years).

CoAs were determined for pairs of noncalf individuals of known sex sighted at least six times per pooled period (this is consistent with previous work [12]). Calves were not included due to their associations being dependent on their mother. Strong associations were defined as greater than twice the average CoA of the study group [29–30].

To test the null hypothesis that individuals associate at random, permutation tests were conducted in SOCPROG by permuting individuals into groups, using 100 flips per iteration, within sampling periods of one day [28]. We tested for between-sampling period association preference/avoidance by comparing the Standard Deviation (SD) of the observed half-weight index matrix with the SD expected by chance, i.e. calculated for a total of 10,000 permuted matrices (which was enough permutation to stabilize the P-value). The P-value was calculated as the number of times the SD from the permuted data was less than the observed SD. We rejected the null hypothesis when the SD of the observed half-weight indices were higher than the permuted SD. This means there was more variation in the real indices than expected by chance, i.e. higher and lower CoAs which can be interpreted as preferred and avoided associations, respectively [28]. For nonrandom associations, Mantel tests were conducted to examine whether differences in association occur between classes (*e*.*g*. sex, age class, cluster, residency, where appropriate).

The correlation coefficient (CC) between the true and calculated association indices [30–31] was used to infer on the reliability of the results and if the data was a reliable representation of the social system. The statistical power of the permutation test was estimated with the Social differentiation (S, a measure of the variability of the associations where 0 is homogenous and over 1 indicates considerable diversity) and the mean number of observed associations per individual (H), giving that S^2^xH>5 suggests sufficient power [31]. The precision (Standard Errors, SE) of the estimate of the Social Differentiation and CC was estimated with bootstrap techniques (100 replications).

Non-parametric multidimensional scaling (nMDS) and hierarchical agglomerative cluster analyses were used to investigate cluster stability/changes on LBB and GBB. In nMDS plots individuals strongly associated will be plotted together, and weakly associated farther apart [28]. The stress of the nMDS indicates how representative the 2D plot is of the data, with lower stress values indicating a better representation. The number of dimensions was increased until the stress fell below 0.10, and the starting position was set to random. A network diagram was drawn based on this nMDS plot arrangement in which nodes representing individuals are connected by links, whose thickness indicate their CoA values. The average-linkage method was used for the hierarchical cluster analysis and produced a dendogram where individuals are on one axis and their CoA on another [28] and a cophenetic correlation coefficient (which is a measure of how well the dendogram matches the matrix of association indices) of > 0.80 indicates a good match to the association matrix [30].

#### Breeding population size

To calculate breeding population size of the original LBB community, we estimated the age of animals during each of the three time periods used for social structure analysis (2007–2009, 2010–2012, 2013–2015). Ages were estimated through a combination of observational data and generalized age-class ranges. Because Atlantic spotted dolphins gain spots as they age, age estimates can be made based on the spotting and color patterns of individuals. In this study, age ranges were based on identified age class at the time they were first observed in the field.

Females typically achieve first parturition during the mottled age class [6]. The exact age at which males reach sexual maturity is unknown in this species, therefore, we first assumed that males reach sexual maturity at the same age as females. We considered all animals in the mottled and fused age classes as contributors to the breeding population, regardless of other factors (*i*.*e*., sex or proof of reproductive success). We considered the estimate including all mottled animals to be non-conservative because it may include more animals, specifically males, that have not actually contributed offspring to the population and may result in an overestimate of the breeding population. Previous paternity analysis indicated that males may not successfully breed until they reach the fused age class [15]. Therefore, we used a more conservative approach that was less likely to overestimate the breeding population size by excluding mottled males as contributors to the breeding population. The operational sex ratio (OSR) was calculated as the proportion of breeding males out of all breeding males and females. We tested whether the OSR was different from an expectation of equal sexes using chi-square analysis.

### Genetic relatedness

The exodus of such a large proportion of a breeding group may have serious implications for the genetic health of the remaining population. To better understand whether the remaining individuals were at an increased risk of inbreeding, we determined whether the mean relatedness among the remaining individuals decreased with the loss of animals to another location and compared it to the mean relatedness of the original group and the animals that relocated.

We used microsatellite genotypes generated from previous work [15,32] to determine coefficients of relatedness (*r*-values) among pairs of individuals using RELATEDNESS v. 5.0.8 [33]. Values range from -1–1 with negative values indicating that relatedness between two individuals is less than expected between individuals chosen at random and positive values indicate some degree of relatedness. When generating relatedness values prior to the move, the known maternal relatives were removed to reduce bias and estimates were jackknifed over loci to generate standard errors. After the move we generated relatedness values both with and without maternal relatives in the analysis to assess the impact of the reduction in sample size.

Allele frequencies were generated using residency groupings for the three different time periods. Pairwise *r*-values were estimated for all pairs of individuals with genotype data. Average *r*-values within families, clusters and resident groups were estimated including all individuals except in cases of known maternal relatives that were excluded from the calculation.

### Habitat and oceanographic data—Interannual trends

**Fig 2:**
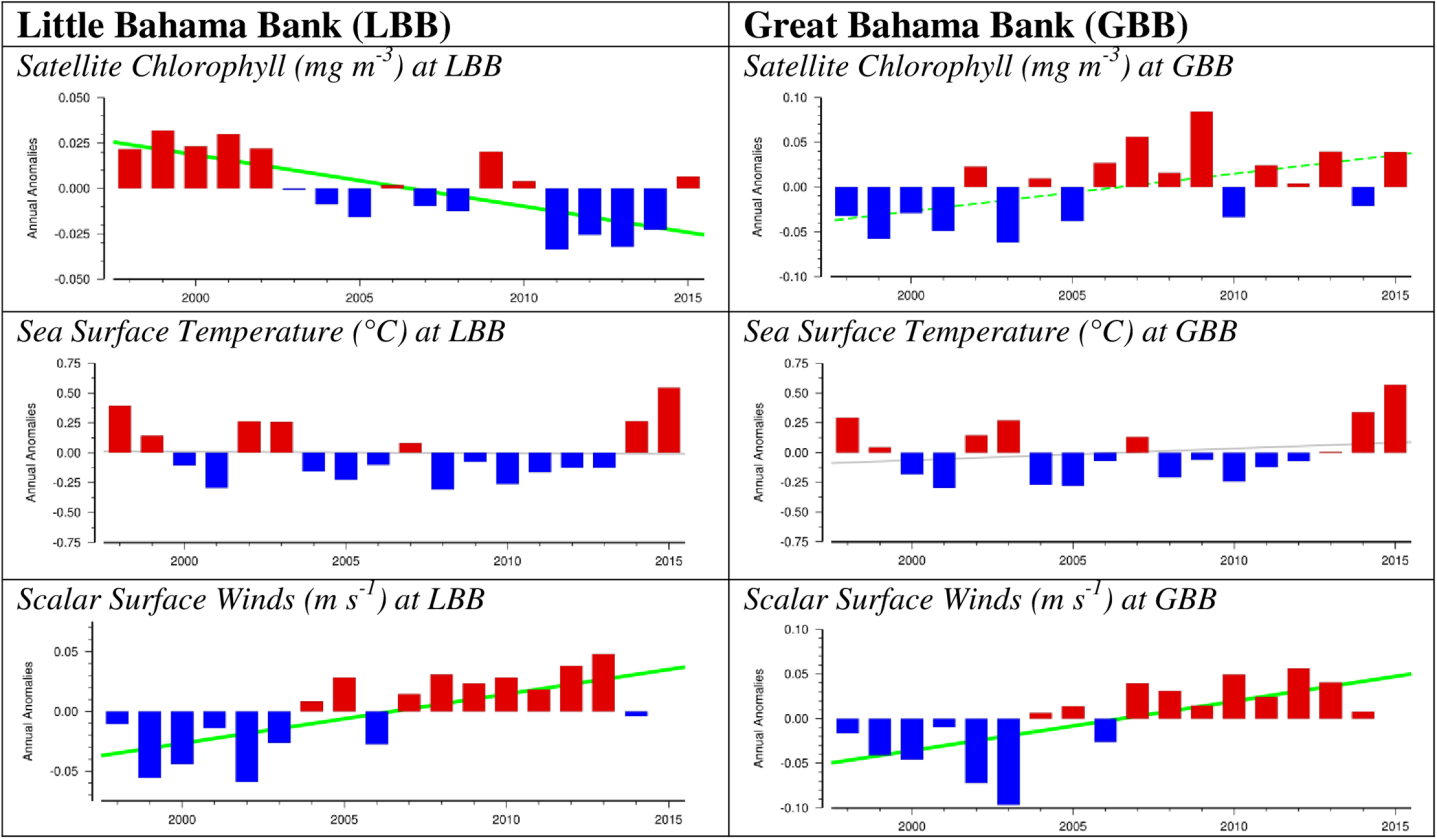
COPEPOD data for the Bahamas. Data includes Satellite Chlorophyll, Sea Surface Temperature, and Scalar Surface winds on Little Bahama Bank (LBB)–left panels) and Great Bahama Bank (GBB- right panels).

To examine and compare trends in sea surface temperatures (°C), productivity as estimated with surface chlorophyll *a* concentrations (mg m^-3^), and surface scalar wind speeds (m s^-1^) between LBB and GBB in the northern Bahamas, we used data and time-series analytical tools from the Interactive Time-series Explorer toolkit of the Coastal and Oceanic Plankton Ecology, Production and Observation Database, COPEPOD http://www.st.nmfs.noaa.gov/copepod [34] (Fig 2). COPEPOD is a global database of plankton survey data, supporting data products, and time series data extraction and exploration tools [35–36] hosted by the National Marine Fisheries Service of the U.S. National Oceanic and Atmospheric Administration.

Because contiguous phytoplankton, zooplankton, and *in situ* chlorophyll data were not available for either LBB or GBB, we used satellite surface chlorophyll *a* concentrations at LBB and GBB as a proxy for lower tropic level food availability in these areas. Chlorophyll is a rough indicator of phytoplankton concentration, and thus food available to the zooplankton and upper trophic levels (*e*.*g*., fish, sea birds, and marine mammals).

Using the geographic coordinates for the LBB and GBB sub-areas (Fig 1), we used COPEPOD’s time series toolkit (http://www.st.nmfs.noaa.gov/copepodite/toolkit) to extract and generate spatially-averaged time series of sea surface temperature (from NOAA’s Optimum Interpolation Sea Surface Temperature data set, OISST version 2, https://www.ncdc.noaa.gov/oisst), satellite-based surface chlorophyll *a* concentration (from NASA’s SeaWiFS/MODIS-A data set, https://oceandata.sci.gsfc.nasa.gov), and surface wind speed (from the International Comprehensive Ocean-Atmosphere Data Set, ICOADS release 2.5, https://rda.ucar.edu/datasets/ds540.0). All of the time-series in our analysis were truncated to start in 1998, as this is the first year that SeaWiFS satellite chlorophyll data were available for LBB and GBB.

Seasonally corrected annual anomalies of sea surface temperature and chlorophyll data from 1998–2015, and of surface wind speed from 1998–2014 (the last year data were available) were calculated for the LBB and GBB areas using the COPEPOD toolkit. This calculation method is described in [35], with additional documentation also available online at http://www.st.nmfs.noaa.gov/copepod/time-series-methods/.

Interannual seasonally corrected annual anomalies in sea surface temperature, chlorophyll data and surface wind speed were analyzed from 1998–2012, to examine trends through the final year the 52 spotted dolphins involved in the move to GBB were sighted on LBB. Separately, interannual seasonally corrected annual anomalies in sea surface temperature and chlorophyll data were analyzed from 2013 to 2015 to examine for trends from the first year the 52 spotted dolphins involved in the move to GBB were sighted on GBB. However, a similar analysis on annual anomalies in wind speed was not conducted as these data were only available through 2014.

Inasmuch as many spotted dolphins feed both on the sandbank and also off the deep water (300m) edge of the bank (in evening hours), we also conducted a microanalysis of seasonally corrected annual anomalies of sea surface temperature, chlorophyll data and surface wind speed both on the shallow sandbank and off adjacent deep waters of the sandbank (see Fig 2) for both LBB and GBB for those factors found to have significant overall trends in LBB and GBB.

## Results

Between Fall 2012 and Spring 2013, a total of 52 spotted dolphins (25 males = 15 adults 5 juveniles, 5 calves and 27 females = 16 adults, 8 juveniles, 3 calves) that had been resident (in three distinct clusters) on LBB up to the Fall of 2012, moved to GBB. We compared changes in their group size and describe the interactions between the displaced dolphins and resident dolphins on GBB.

### Encounters and group size on LBB and GBB

The number of encounters varied across years and locations of both Atlantic spotted dolphins and bottlenose dolphins (Table 2). Effort to survey in both study sites over the years was successful, although the number of encounters varied between location and species.

**Table 2:** Number of encounters by species and location.

Year	LBB SP on LBB	LBB SP on GBB	LBB/GBB SP on GBB	GBB SP on GBB	LBB BN only	LBB Mixed	GBB BN only	GBB Mixed
2013	16	9	2	1	3	2	2	3
2014	7	10	9	18	5	11	1	6
2015	7	3	6	8	1	11	3	1
Totals	30	22	17	27	9	24	6	10

SP = spotted dolphins; BN = bottlenose dolphins; Mixed = bottlenose and spotted dolphins.

LBB = Little Bahama Bank; GBB = Great Bahama Bank.

Mean group size changed over the years in the different locations by different *Community ID* (Table 3). Mean group size of LBB dolphins on LBB remained similar over the three-year period (Kruskal-Wallis H = 0.21675, df = 2, p = 0.89729). Although mean group size for LBB dolphins on GBB decreased over the three-year period the decrease was not significant (Kruskal-Wallis H = 4.48571, df = 2, p = 0.10615). Mean group size for LBB/GBB dolphins on GBB was also not significantly different across the three-year period (Kruskal-Wallis H = 0.39125, df = 2, p = 0.82232). There was no significant difference in group size for *Community ID* across 2013 (Kruskal-Wallis H = 2.5, df = 2, p = 0.28650) or across 2015 (Kruskal-Wallis H = 3.8044, df = 2, p = 0.14924) but there was a significant in 2014 across Community IDs (Kruskal-Wallis H- 6.69082, df = 2, p = 0.03525).

**Table 3:** Group size for spotted dolphins by location and community ID.

YEAR	LBB spotted dolphin group size on LBB	LBB spotted dolphin group size on GBB	LBB and GBB spotted dolphin group size on GBB
**2013**	11.81± 8.44	14.67 ± 10.43	22.50 ± 9.63
**2014**	9.86 ± 7.31	8.50 ± 3.10	21.78 ± 12.40
**2015**	10.14 ± 6.24	3.33 ± 1.25	18.00 ± 9.73

Mean (SD) group size for spotted dolphins by location (Little Bahama Bank–LBB, Great Bahama Bank–GBB) and *Community ID* (LBB dolphins on LBB, GBB, or LBB/GBB mixed species on GBB).

### Site fidelity on GBB

**Fig 3:**
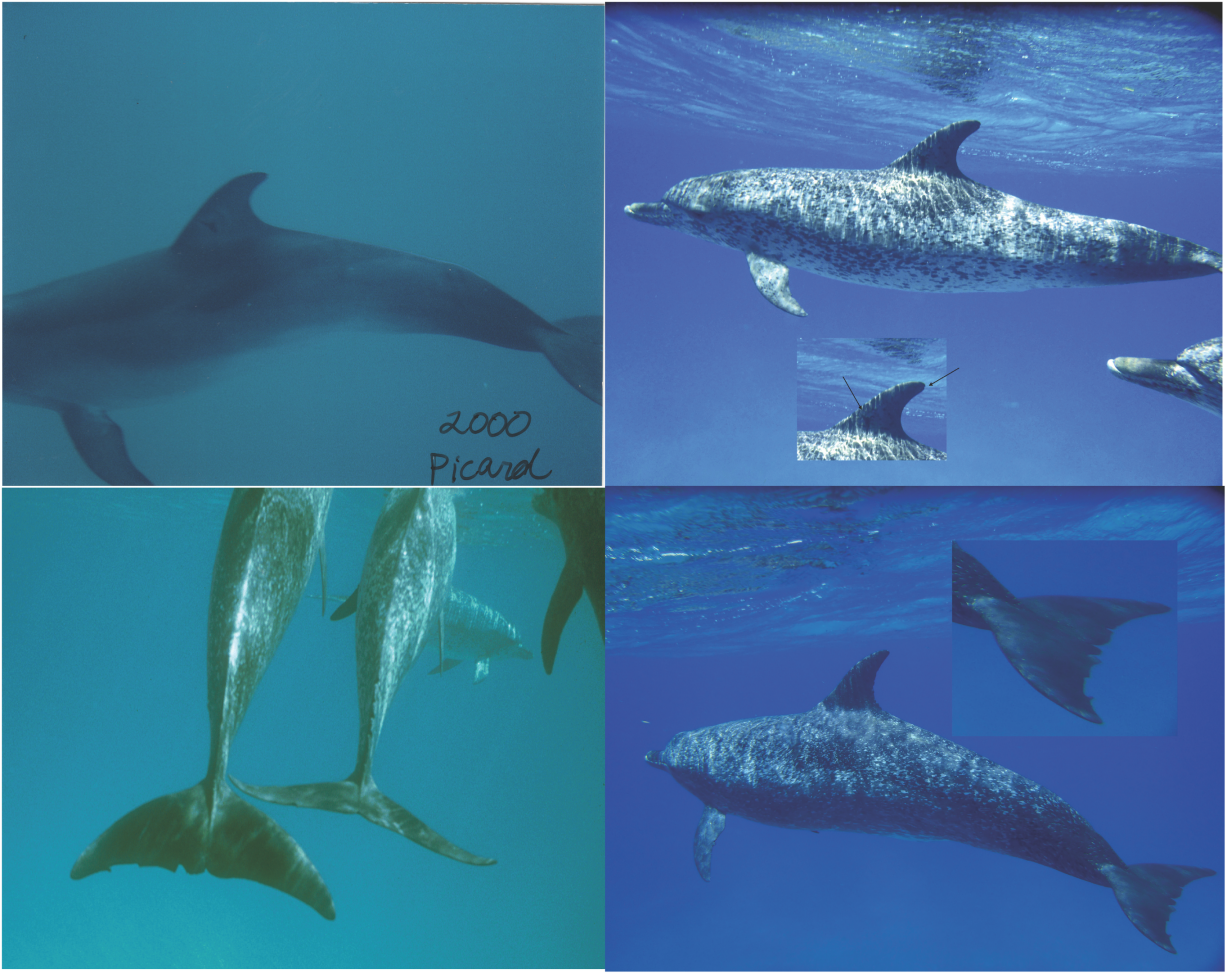
Identification of two spotted dolphins on GBB over a decade. Top: Female Atlantic spotted dolphin Picard with a distinct insignia mark, in 2000 as a juvenile on Great Bahama Bank (GBB) and again in 2013 as a young adult mottled on GBB. Black arrows denote dorsal fin mark and nick in tip of fin. Bottom: Male adult fused Atlantic spotted dolphin, Salinger, with distinctive fluke, taken on GBB in 1998 and again in 2013 on GBB. Photo credit: Wild Dolphin Project.

Site fidelity was documented on GBB with at least two dolphins over at least a four-year period, Salinger (adult male–observed 1998–2003) and Picard (juvenile female– 2000–2003) both resighted in the summer of 2013 by WDP on GBB. This suggests that there is site fidelity and residency of at least some (and likely more) spotted dolphins on GBB over the decades (Fig 3).

### Cluster changes on LBB

After the 2015 season, tallies of displaced dolphins included not only the entire *Central* cluster (45 individuals) but also 5 individuals from the *Northern* and 2 from the *Southern* cluster. A few unusual disruptions to matrilineal relationships from the *Northern* and *Southern* clusters were also noted.

*Northern* cluster–A total of 5 individuals moved to GBB from LBB. One young adult female (Burgundy) moved to GBB while her mother and multiple siblings remained on LBB (Brush/Palette, Brat) and one adult female (Tyler) moved to Bimini leaving her female juvenile offspring (Tristan). One adult male (Liney) and 2 young adult males (Picasso and Sunami) also moved to GBB. *Central* cluster—all 45 individuals (37 non-calves, 8 calves) moved to GBB from LBB. *Southern* cluster—One juvenile male (Infinite) moved to GBB while her mother remained on LBB, and one juvenile female (Marble-matriline unknown) moved to GBB from LBB.

### Reproduction and health

Of the LBB spotted dolphins that migrated to GBB, average pregnancy rates and calving rates have remained within normal ranges (1–10 calves/yr, [6]) and are as follows: (2013:11 calves, 2014: 8 calves 2015: 1 calf). Based on our observations of body characteristics, the physical health of the dolphins appeared uncompromised (*e*.*g*., no dolphin appeared emaciated).

### Social structure results

The total number of encounters, spotted dolphin individuals (male/females) and CoA statistics (including mean, SD, CV, S, CC, H, S^2^xH and P value) for all pooled years are given in Tables 4 and 5. Due to the different locations after the displacement, the 2013–2015 data are split into two datasets (GBB vs. LBB). For all these datasets the statistical power to reject the null hypothesis of no preferred companions was good with S^2^xH > 5. The correlation coefficient (CC) was close to or above the 0.80 criterion of a good representation. This indicates the data are reliable and a good representation of the true social system (Table 4).

**Table 4:** Social differentiation, representation and statistical power of permutation tests.

Year	S+SE	H	CC+SE	S^2^xH
post-hurricane*	0.52 +0.07	162.43	0.70 + 0.03	44.4
2007–2009	0.42 ± 0.06	245.5	0.70 ± 0.04	43.92
2010–2012	0.67 ± 0.04	235.79	0.84 ± 0.03	105.85
2013–2015 LBB	0.59 ± 0.12	49.87	0.86 ± 0.04	17.59
2013–2015 GBB	0.73 ± 0.05	88.45	0.72 ± 0.05	63.42

Social differentiation (S) + Standard Error (SE), Number of associations per individual (H), Correlation Coefficient (CC) +SE. S>0.50 high social differentiation, CC>0.80 good representation, S^2^xH >5 good ability to reject null hypothesis of no preferred companions.

* Data from post-hurricane years (2005–2007) is given for reference from [14].

**Table 5:** CoA statistics for pooled years by encounters and individuals.

Year	Enc	Individuals	Mean	SD	CV
post-hurricane*	91	47 (23M/24F)	0.24 + 0.16	0.16(real)/0.15(random)	0.66(real)/0.61(random)
2007–2009	133	51(24M/27F)	0.23 ± 0.14	**0.14(real)/0.13(random)**	**0.61(real)/0.56(random)**
2010–2012	168	57(23M/34F)	0.20 ± 0.16	**0.16(real)/0.14(random)**	**0.80(real)/0.73(random)**
2013–2015 LBB	51	15 (2M/13F)	0.33 ± 0.23	0.23(real)/0.23(random)	0.69(real)/0.69(random)
2013–2015 GBB	82	62 (29M/33F)	0.17 ± 0.17	**0.18(real)/0.15(random)**	**1.02(real)/0.87(random)**

Number of encounters (Enc), number of individuals (M = male, F = female), CoA statistics: Mean, Standard Deviation (SD) and Coefficient of Variation (CV) from permutation tests. SD and CV (real) significantly higher than random = nonrandom associations (in bold).

* Data from post-hurricane years (2005–2007) is given for reference from [14].

#### Social structure 2007–2009

The annual immigration rate remained low at 4.3 noncalf individuals per year. Permutation tests revealed nonrandom associations (SD and CV Table 5 p <0.001). The results were consistent with the post-hurricane years [14]: the mean CoA was similar as well as the social differentiation remaining lower (Tables 4 and 5) and Mantel tests (Table 6) found within class CoA to be higher than between class CoA for cluster (t = 5.05, r = 0.36, p < 0.001) and sex (t = 6.68, r = 0.16, p< 0.001). Within class CoA for age class were found to be higher than between (Table 6, t = 2.63, r = 0.12, p = 0.01) which was not found post-hurricane (although when broken down by sex, there were still some differences in CoAs in relation to age [14]). Social differentiation by cluster was low (*Central*, 38 individuals: S = 0.29 ± 0.05; *Southern*, 6 individuals: S = 0.00 ± 0.16; *Northern*, 7 individuals: S = 0.22 ± 0.09)

**Table 6:** Mantel tests for association indices within and between cluster, sex and age class for each pooled year set.

Year	Within cluster	Between cluster	Within sex	Between sex	Within age	Between age
2007–2009	**0.30**	0.18	**0.26**	0.21	**0.25**	0.22
2010–2012	**0.30**	0.11	**0.23**	0.18	**0.20**	0.19
2013–2015 LBB	NA	NA	NA	NA	NA	NA
2013–2015 GBB	0.30*	0.06*	**0.19**	0.17	**0.20**	0.16

Bold indicates significantly higher CoA.

* for the 2013–2015 GBB set cluster means residency (MGBB vs. RGBB).

#### Social structure 2010–2012

The annual immigration rate remained low at 1 non-calf individual per year. Permutation tests revealed nonrandom associations (SD and CV Table 5 p = 0.0001). The mean CoA was similar to 2007–2009, however social differentiation increased, edging closer to what was seen pre-hurricane [12]. Mantel tests (Table 6) found within class CoA to be higher than between class CoA for cluster (t = 10.85, r = 0.56, p = 0.0), sex (t = 3.36, r = 0.10, p = 0.01), and slightly significantly higher for age class (t = 1.93, r = 0.09, p = 0.04). Social differentiation by cluster was low (*Central*, 37 individuals: S = 0.30 ± 0.05; *Southern*, 13 individuals: S = 0.34 ± 0.13; *Northern*, 7 individuals: S = 0.14 ± 0.10).

#### Social structure 2013–2015 on LBB

Annual immigration rates remained low at 1.3 non-calf individuals per year. Permutation tests revealed random associations (SD and CV Table 5 p >0.35). The mean CoA increased compared to previous years (Table 5) and social differentiation remained near 2010–2012 levels (Table 4).

#### Social structure 2013–2015 on GBB

**Fig 4:**
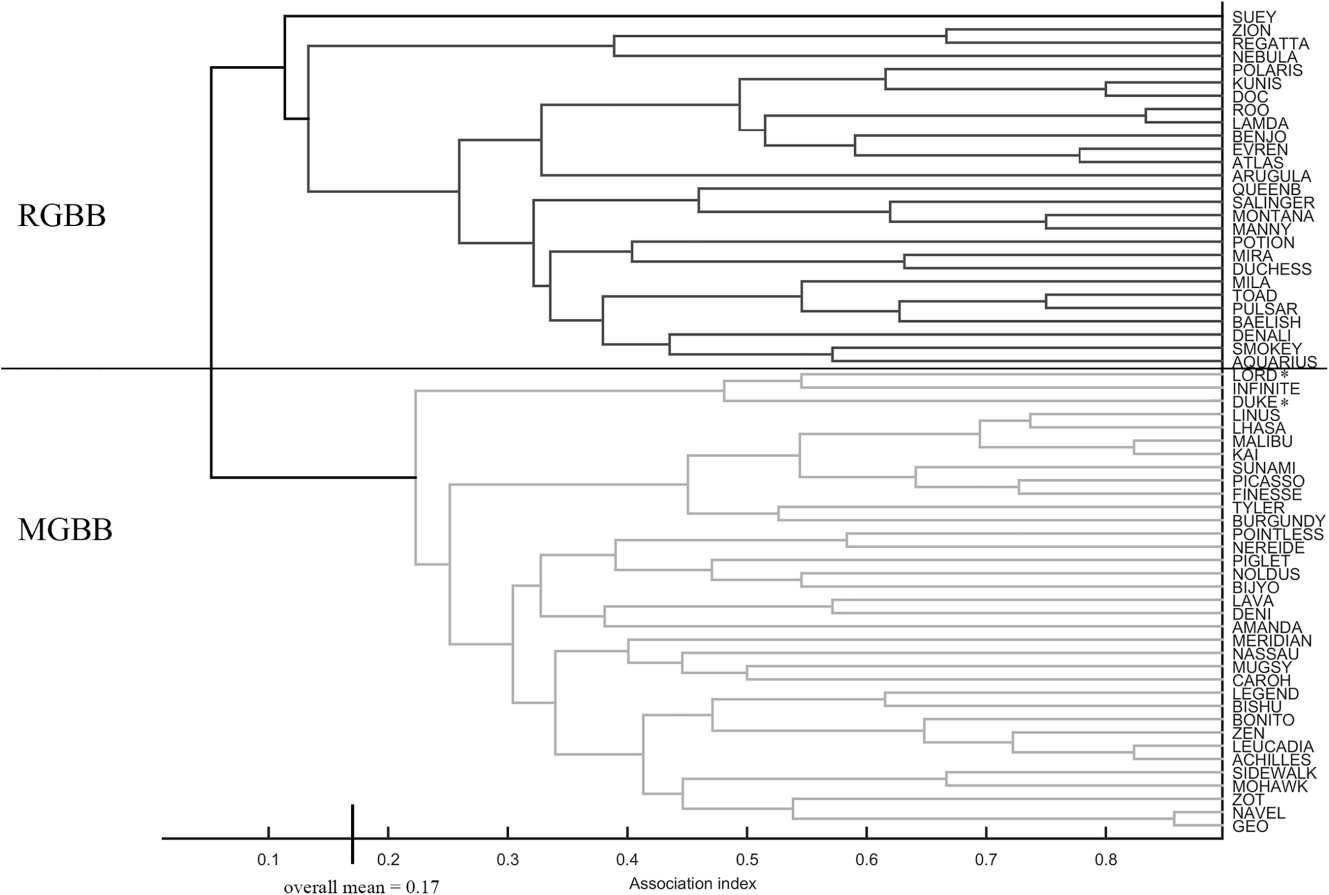
Cluster dendogram for GBB 2013–2015. Average linkage method, CCC = 0.83, modularity 0.34. RGBB (resident to GBB), MGBB (moved to GBB). *Lord and Duke are RGBB animals that have been grouped with the MGBB in the cluster diagram, but are grouped with RGBB animals in the network diagram.

**Fig 5:**
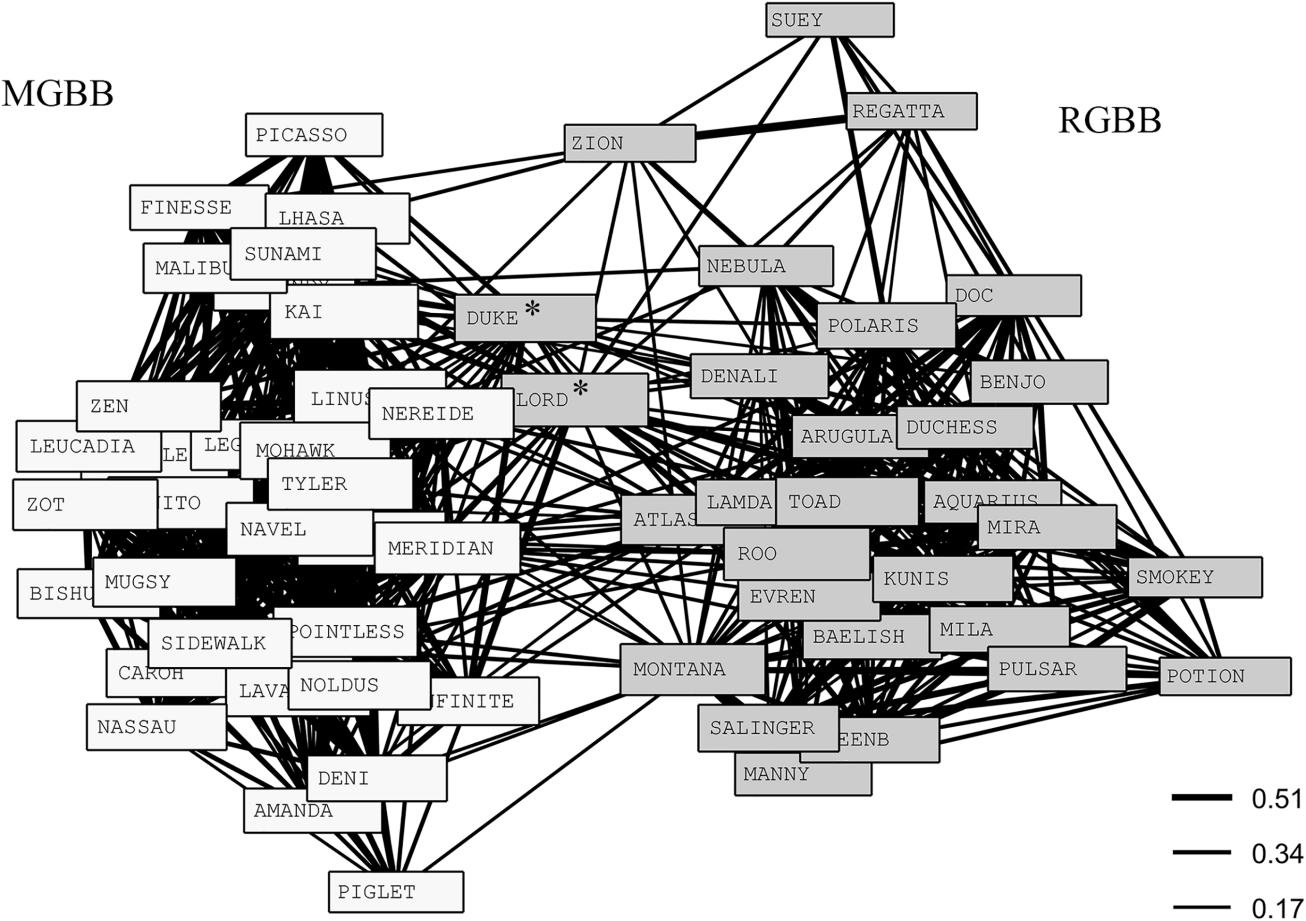
Network diagram for GBB 2013–2015. Based on nMDS plot (4 dimensions, stress = 0.097). Legend indicates CoA values for line strength: 0.17 overall mean, 0.34 twice the overall mean (strong associations), 0.51 (three times the overall mean). * indicates two RGBB animals that are grouped with RGBB here (although on edge of cluster), but have a lot of links to MGBB cluster, and were grouped with MGBB in cluster analysis.

Annual immigration rates are unknown for GBB location. Permutation tests revealed nonrandom associations (SD and CV Table 5 p <0.001). The mean CoA was lowest and social differentiation was highest and closest to that seen in long-term pre-hurricane analyses on LBB [12]. Social differentiation for just MGBB dolphins (there was no difference between clusters, CoA 0.34 *vs*. 0.29 t = 1.33, r = 0.10, p = 0.11) revealed homogenous associations (S = 0.00 ± 0.14), whereas RGBB dolphins had low differentiation (S = 0.27 ± 0.12). For all dolphins on GBB Table 6 shows Mantel tests found within class CoA to be higher than between class CoA for residency (MGBB *vs*. RGBB) (t = 29.80, r = 0.67, P = 0.00), sex (t = 2.39, r = 0.05, p = 0.03), and age (t = 4.34, r = 0.10, p = 0.002). Fig 4 (S1 Fig) and Fig 5 (S2 Fig) show a cluster dendogram and network diagram (based on nMDS plot) respectively, that support the Mantel results concerning residency and the extreme separation between the MGBB dolphins and those RGBB dolphins. In the network diagram there seem to be three individuals (Zion (female), Lord and Duke (males)) that appear to link the two clusters, and in the cluster dendogram, two of these RGBB animals (Lord and Duke) are actually clustered with the MGBB animals.

#### Social structure male alliances

The presence of adult (mottled or fused) male spotted dolphins remaining on LBB after 2012 was greatly reduced from 28 to only five; 15 moved to GBB and eight have been lost (not seen anywhere since 2012). It should be noted that the number of males remaining in the alliance analysis is less than the actual number of males physically remaining in the respective communities because of the requirement of ≥6 sightings per individual. Most males in the area (*Central* cluster) moved to GBB. Very few males were left on LBB, and only two of these males had enough sightings to be included in analyses. These two were adult immigrants (in 2009) and had a CoA of 0.95, much higher than twice the 0.33 overall mean; however the overall associations were found to be random, so care should be taken in assigning this as an alliance. No alliances that were on LBB prior to the move remained on LBB.

After the immigration event, alliances between male spotted dolphins on GBB were observed, but there were no strong associations between MGBB and RGBB males. Indeed, there was little observed integration between the MGBB and RGBB spotted dolphins. Therefore, CoA levels for male alliances were not based on the overall male-male mean, as it would have been artificially low due to their low interaction, not actually preferred avoidance of specific males. Thus an alliance was defined as twice the mean for each cluster (MGBB male-male mean = 0.37, RGBB male-male mean = 0.28). Using this modified metric, MGBB had four alliances and RGBB had three alliances.

Two of the four MGBB alliances were previously known LBB alliances that survived the move, with slight changes. Linus, Kai and Lhasa were a speckled trio that formed after the 2004 hurricanes on LBB, with Kai and Lhasa the primary pair. On GBB, Kai and Malibu (who was previously in a different alliance and whose partner was subsequently lost), were now the primary pair (CoA = 0.82). Linus, Kai and Lhasa had a very strong association with each other (CoA 0.74–0.76) and Linus and Lhasa have less strong associations with Malibu (CoA 0.60–0.67), making it difficult to determine the exact structure of their alliance. They are now all mottled/fused individuals. Sunami and Picasso were another pair that formed on LBB after the hurricanes (both speckled at the time). On GBB Picasso and Finesse (no known previous alliance) are now the primary pair (CoA = 0.73, just below the 0.74 cut off) with Sunami a possible third member (CoA = 0.62–0.67), now all mottled/fused individuals. Interestingly both of these alliances formed after the hurricanes between speckled individuals, which was the first documentation of juvenile alliance level associations in that community [14].

A third MGBB alliance formed between speckled individuals: Leucadia and Achilles (CoA = 0.82) with both Bonito (speckled) and Zen (mottled) as strong partners (CoA = 0.67–0.78 with the primary pair Leucadia and Achilles, and 0.50 between Bonito and Zen), again making it difficult to determine the exact structure. The final fourth MGBB alliance is a pair of fused individuals: Navel and Geo (CoA = 0.86, the strongest CoA observed). Both of these individuals where not strongly associated with any particular males prior to the move. It is interesting to note that two of the four alliances of MGBB involve four individuals instead of the normal two or three.

There were three RGBB male alliances. Evren and Atlas (both speckled, CoA = 0.78) was the primary pair, with Benjo as the odd male (speckled, CoA = 0.56–0.63) and a possible fourth member Doc (mottled, CoA = 0.53–0.59). Another alliance was Montana and Manny as the primary pair (CoA = 0.75) with Salinger as the odd male (CoA = 0.53–0.71), all fused individuals. The last alliance is Toad and Pulsar (CoA = 0.75) with Baelish as the odd male (CoA = 0.59–0.67), all mottled individuals.

### Breeding population estimates

The breeding population (mottled and fused males included) on LBB was reduced from 45 potential breeding contributors (2010–2012) to 24 (2013–2015) as a result of the move of animals to GBB (Table 7). If only fused males are considered contributors with mottled and fused females, the breeding group was reduced from 34 individuals to 23. The *Northern cluster* lost three males, which was a substantial proportion of the males in the cluster (75%) given its small size. The *Central* social cluster was reduced to zero total animals. The *Southern cluster* actually increased from 4 breeding males in the pre-move period (2010–2012) to 5 males (1 mottled and 4 fused) in the post-move period (2013–2015; Table 7). We have observed 57 total resident animals on GBB. Therefore, the total group of animals on GBB consists of at least 112 animals following the move, including new calves that have been born into the groups.

**Table 7:** Operational sex ratio for all years and locations.

Residency	Base 2007–2009				Pre-Move 2010–2012					Post-Move 2013–2015			
	TA	TB_mf_ (TB_f_)	M:F_mf_ (M:F_f_)	OSR_mf_ (OSR_f_) §	TA	TB_mf_ (TB_f_)	M:F_mf_ (M:F_f_)	OSR_mf_ (OSR_f_) §		TA	TB_mf_ (TB_f_)	M:F_mf_ (M:F_f_)	OSR_mf_ (OSR_f_) §
RLBB													
North		4 (3)	2:2 (1:2)	0.50 (0.33)		6 (3)	4:2 (1:2)		0.67 (0.33)		2 (2)	1:1 (1:1)	0.50 (0.50)
Central		19 (18)	7:12 (6:12)	0.37 (0.33)		22 (19)	10:12 (7:12)		0.47 (0.37)		0 (0)	0:0 (0:0)	0.00 (0.00)
South		13 (13)	4:9 (4:9)	0.31 (0.31)		17 (17)	4:13 (4:13)		0.24 (0.24)		22 (21)	5:17 (4:17)	0.23 (0.19)
Total	64	36 (34)	13:23 (11:23)	0.36 (0.32)	75	45 (39)	18:27 (12:27)		0.40 (0.31)	44	24 (23)	6:18 (5:18)	0.25 (0.22)
MGBB										47	31 (27)	14:17 (10:17)	0.45 (0.37)
RGBB										57	23 (18)	10:13 (5:13)	0.43 (0.28)
Total GBBǂ										112	54 (45)	24:30 (15:30)	0.44 (0.33)

Individual residency and age may change between time periods. TA = total number of animals, TB = total number breeding animals, OSR = operational sex ratio, M = male, F = female, mf = using mottled and fused males, f = using fused males only

§ Operational sex ratio reported as the proportion of males.

ǂ Combined MGBB and RGBB.

Prior to the move, the operational sex ratio was skewed towards females among resident animals on LBB at 0.40 but it was not statistically different from a 1:1 ratio (X^2^ = 1.8, p = 0.18, Table 7). The skew towards females was significant when mottled males were not considered (0.32 proportion of males; X^2^ = 6.7, p < 0.01). Operational sex ratios ranged from 0.24 in the *Southern cluster* to 0.37 in the *Central cluster*.

Following the move, the operational sex ratio dropped to 0.25 (0.22 fused males only) among the animals that remained on LBB. The skew in sex ratio was different than the expected 1:1 ratio both when mottled and fused males were included (X^2^ = 6.0, p < 0.05) and when mottled males were excluded (X^2^ = 7.4, p < 0.01). The OSR was reduced to 0.50 in the *Northern cluster* and 0.25 (0.22 fused males only) in the *Southern cluster*.

The OSR among animals that moved from LBB to GBB was 0.45 (0.37 fused males only) that was slightly less than the OSR of the *Central cluster* prior to the move (0.50, 0.37 fused males only). The OSR was not different from 1:1 (mottled and fused males X^2^ = 0.3, p = 0.59; fused males only X^2^ = 1.8, p = 0.18). The resident animals on GBB consisted of 57 total animals, with an OSR of 0.43 (0.28 fused males only), which were not different from 1:1 (mottled and fused males X^2^ = 0.4, p = 0.50; fused males only X^2^ = 3.6, p = 0.06). If mottled and fused males are contributors, the OSR is 0.44, which is not different than 1:1 (X^2^ = 0.7, p = 0.41). However, if only fused males are considered, then the OSR is 0.33, which is statistically skewed towards females (X^2^ = 5.0, p < 0.05).

### Genetic relatedness

We used genotypes from 84 individual Atlantic spotted dolphins that originated from LBB. Prior to the move, the average *r*-value was 0.0920 (SE ± 0.0443, 95% CV = 0.1002). The *Central* cluster had the largest *r*-value indicating the more genetic relatedness than either the *Northern* or *Southern* clusters.

Following the move, only 40 animals with genotypes remained active. Of those, 29 moved to GBB and 11 remained on LBB. The animals that moved had a larger *r*-value than those that stayed. Following the move, the combination of a reduced sample size and the removal of maternal relatives produced lower relatedness values (Table 8). For example, prior to the move, the baseline relatedness within the *Central cluster* was 0.108. The relatedness of 29 animals that moved to GBB was reduced to 0.051 if the maternal relatives were removed. However, if the maternal relatives remain in the analysis, the relatedness was 0.101 that is closer to the pre-move estimate within the *Central* cluster. The same pattern was observed among the animals that remained on LBB with prior *r*-values of 0.075 (*Northern*) and 0.044 (*Southern*) compared to 0.021 of those than remain (regardless of social cluster) if maternal relatives are removed and 0.092 if they remain in the analysis.

**Table 8:** Relatedness of clusters pre move and post move.

Residency	Base All Animals (Pre-move)				Post-Move 2013–2015			
	N	*r*	SE	CV	N	*r*	SE	CV
RLBB								
North	13	0.075	0.041	0.093				
Central	52	0.108	0.048	0.108				
South	19	0.044	0.059	0.133				
Total	84	0.092	0.044	0.1	11	0.021	0.075	0.171
MGBB					29	0.051	0.052	0.118
RGBB					*NA*	—	—	—

N is total number of animals in the group, R is the relatedness coefficient, SE is standard error, and CV is confidence interval. Dolphins on LBB after the displacement (RLBB), those resident to GBB (RGBB), and those that moved from LBB to GBB (MGBB). Values range from -1–1 with negative values indicating that relatedness between two individuals is less than the expected between individuals chosen at random, where positive values indicate some degree of relatedness.

### Habitat and oceanographic trends

#### Interannual trends in sea surface temperature

**Fig 6:**
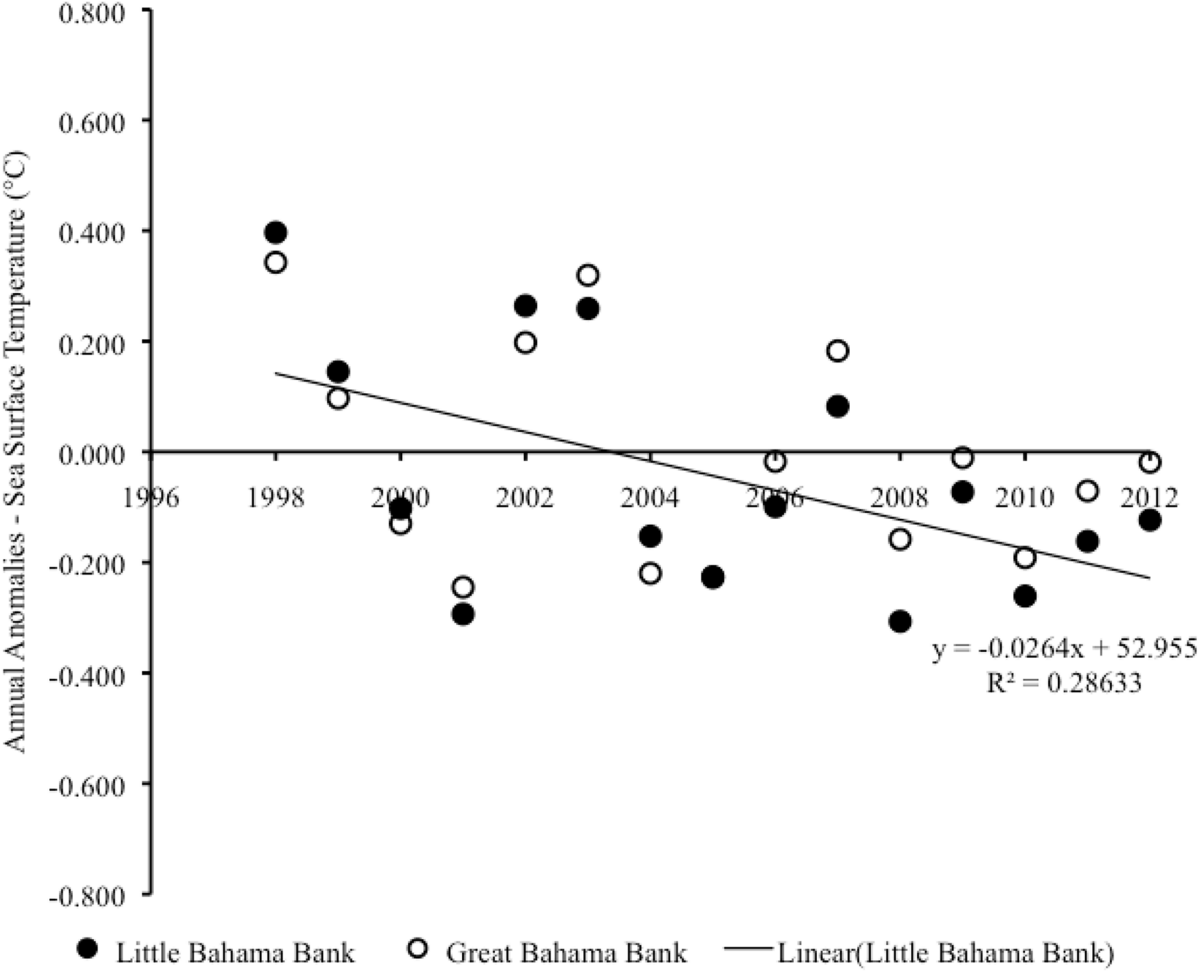
Sea surface temperature anomalies on LBB and GBB. Scatter plot of year vs. annual anomalies in sea surface temperature for the combined shallow and adjacent deep-water areas on LBB and also for GBB from 1998–2012.

**Fig 7:**
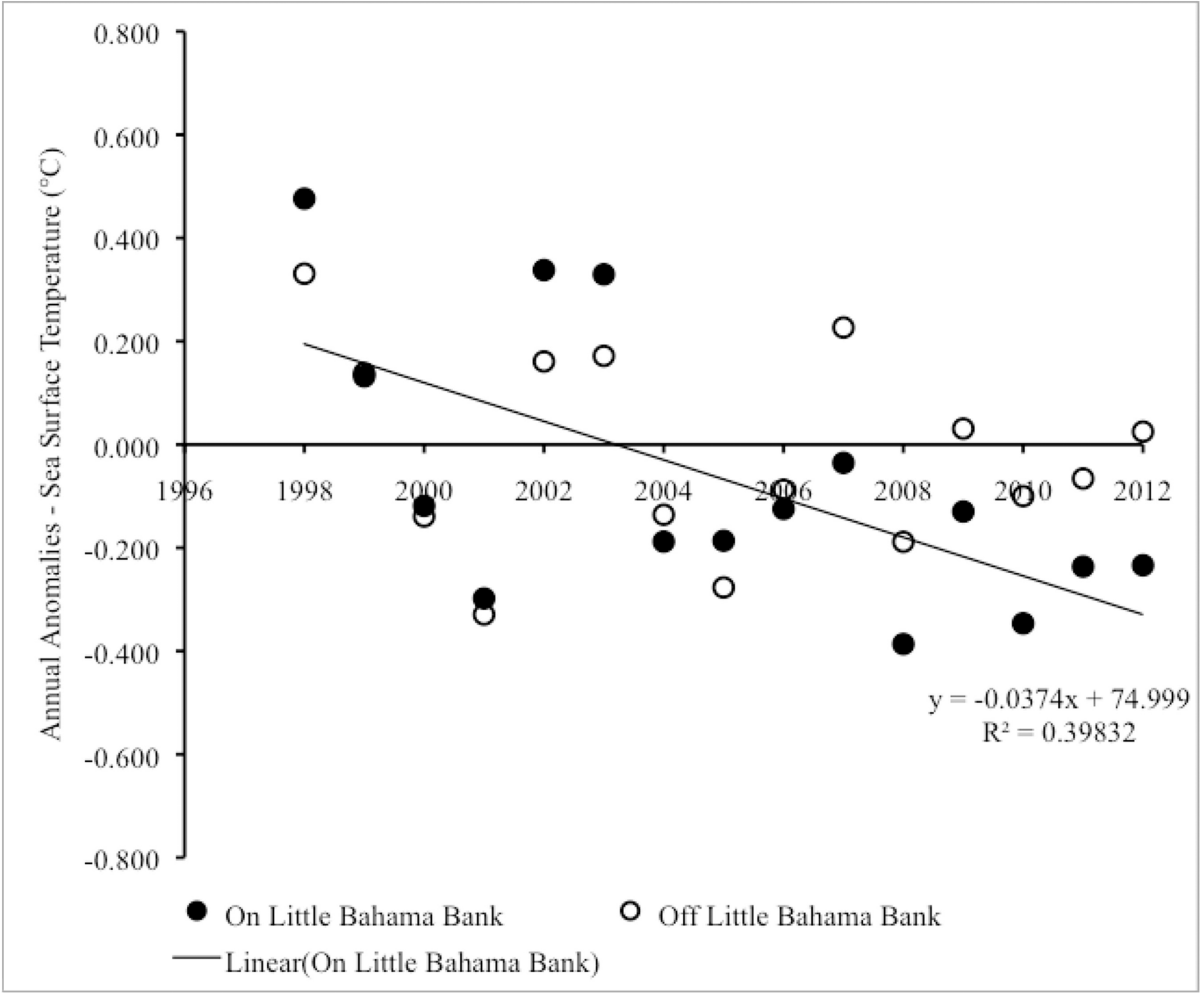
Sea surface temperature anomalies on vs. off LBB. Scatter plot of year vs. annual anomalies in sea surface temperature on LBB vs. off LBB from 1998–2012.

From 1998–2012, there was significant negative trend in annual sea surface temperature anomalies on LBB, which showed a cooling effect (Pearson correlation: r_15_ = -0.54, p = 0.040) (Fig 6). A linear regression analysis revealed that year accounted for a significant proportion of the variance in annual sea surface temperature anomalies during this period (R^2^ = 0.29, F_1,13_ = 5.22, p = 0.040). No similar trend occurred in annual sea surface temperature anomalies on GBB (Pearson correlations: r_15_ = -0.33, p = 0.233) (Fig 6) (S3 Fig). The cooling trend on LBB from 1998–2012 could be traced to significant cooling on the shallow bank (vs. adjacent deep water) of LBB (Pearson correlation: r_15_ = -0.63, p = 0.012) with year accounting for a significant proportion of the variance in annual sea surface temperature anomalies during this period (R^2^ = 0.40, F_1,13_ = 8.61, p = 0.012) (Fig 7) (S4 Fig).

From 2013–2015, the annual sea surface temperature anomalies revealed some warming on both LBB and GBB. However, the trend over the three-year period was not statistically significant in either of these locations (on LBB, Pearson correlation: r_3_ = 0.996, p = 0.060; on GBB, Pearson correlations: r_3_ = 0.995, p = 0.064).

#### Interannual trends in chlorophyll

**Fig 8:**
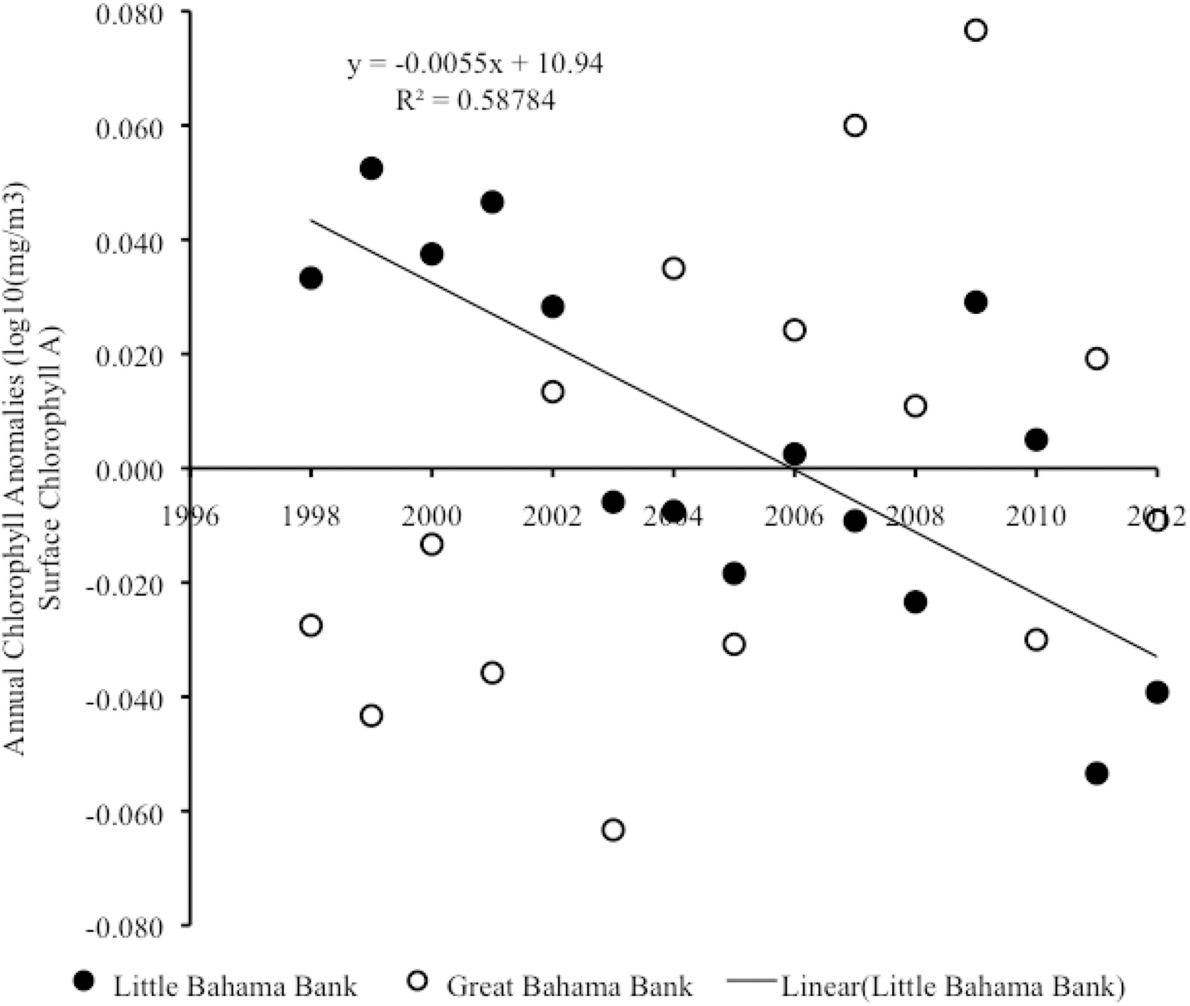
Satellite chlorophyll anomalies on LBB and GBB. Scatter plot of year vs. annual anomalies in chlorophyll production for the combined shallow and adjacent deep-water areas on LBB and also for GBB from 1998–2012. Trend lines, R^2^ and linear regression equations are shown for significant trends.

**Fig 9:**
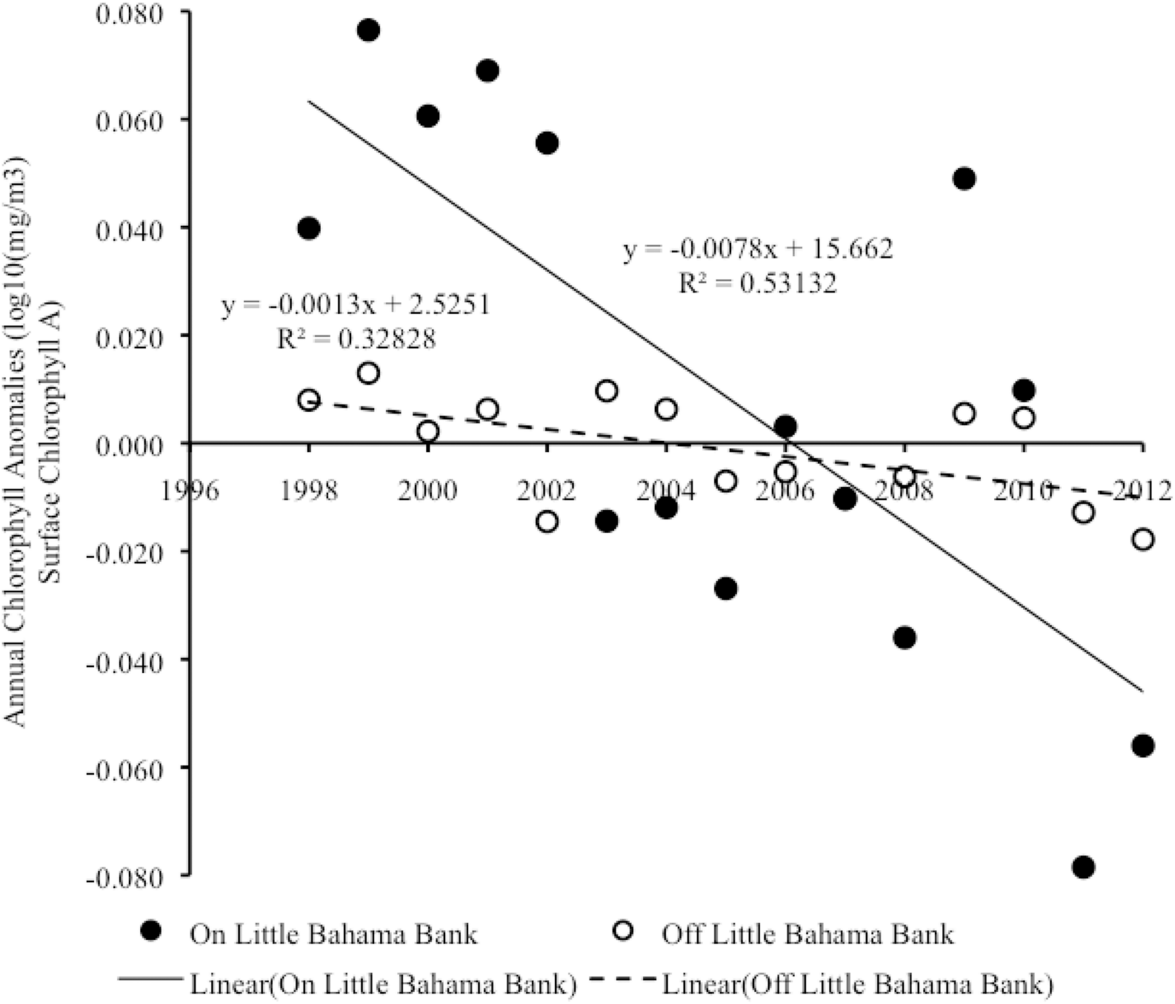
Satellite chlorophyll anomalies on vs. off LBB. Scatter plot of year vs. annual anomalies in chlorophyll production on LBB vs. off LBB from 1998–2012. Trend lines, R^2^ and linear regression equations are shown for significant trends.

From 1998–2012, there was a significant negative linear correlation in year vs. annual anomalies of chlorophyll on LBB (Pearson on: r_15_ = -0.77, p = 0.001), with year accounting for a significant proportion of the variance in annual chlorophyll anomalies (R^2^ = 0.59, F_1,13_ = 18.54, p = 0.001) (Fig 8, S5 Fig). This trend in annual anomalies of chlorophyll was associated with a reduction in chlorophyll production both on LBB (Pearson correlation: r_15_ = -0.73, p = 0.002) and off LBB (Pearson correlation: r_15_ = -0.57, p = 0.026) (Fig 9) (S6 Fig). In both cases, year accounted for a significant proportion of variance in annual anomalies of chlorophyll (On LBB, R^2^ = 0.53, F_1,13_ = 14.74, p = 0.002; Off LBB, R^2^ = 0.33, F_1,13_ = 6.35, p = 0.026). In contrast, over the same period there was no significant relationship between year and annual anomalies in chlorophyll on GBB (Pearson correlation: r_15_ = 0.44, p = 0.098) (Fig 8) (S5 Fig).

From 2013–2015, there was no significant trend in annual anomalies of chlorophyll on either LBB (Pearson correlation: r_3_ = 0.89, p = 0.304) or GBB (Pearson correlation: r_3_ = -0.19, p = 0.877).

#### Interannual trends in scalar surface wind speed

**Fig 10:**
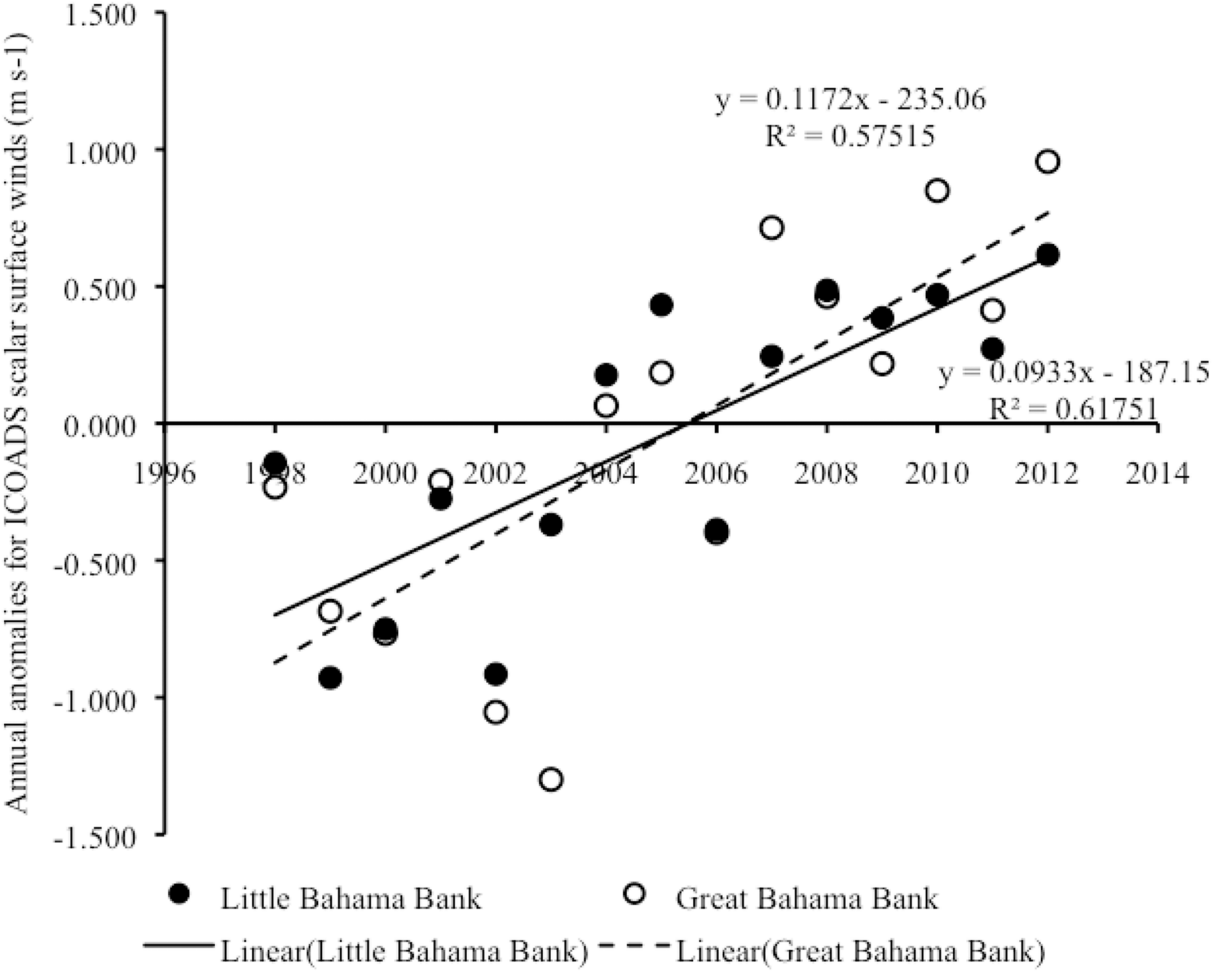
Scalar surface wind anomalies on LBB and GBB. Scatter plot of year vs. annual anomalies in surface winds for the combined shallow and adjacent deep-water areas on LBB and also for GBB from 1998–2012. Trend lines, R^2^ and linear regression equations are shown for significant trends.

**Fig 11:**
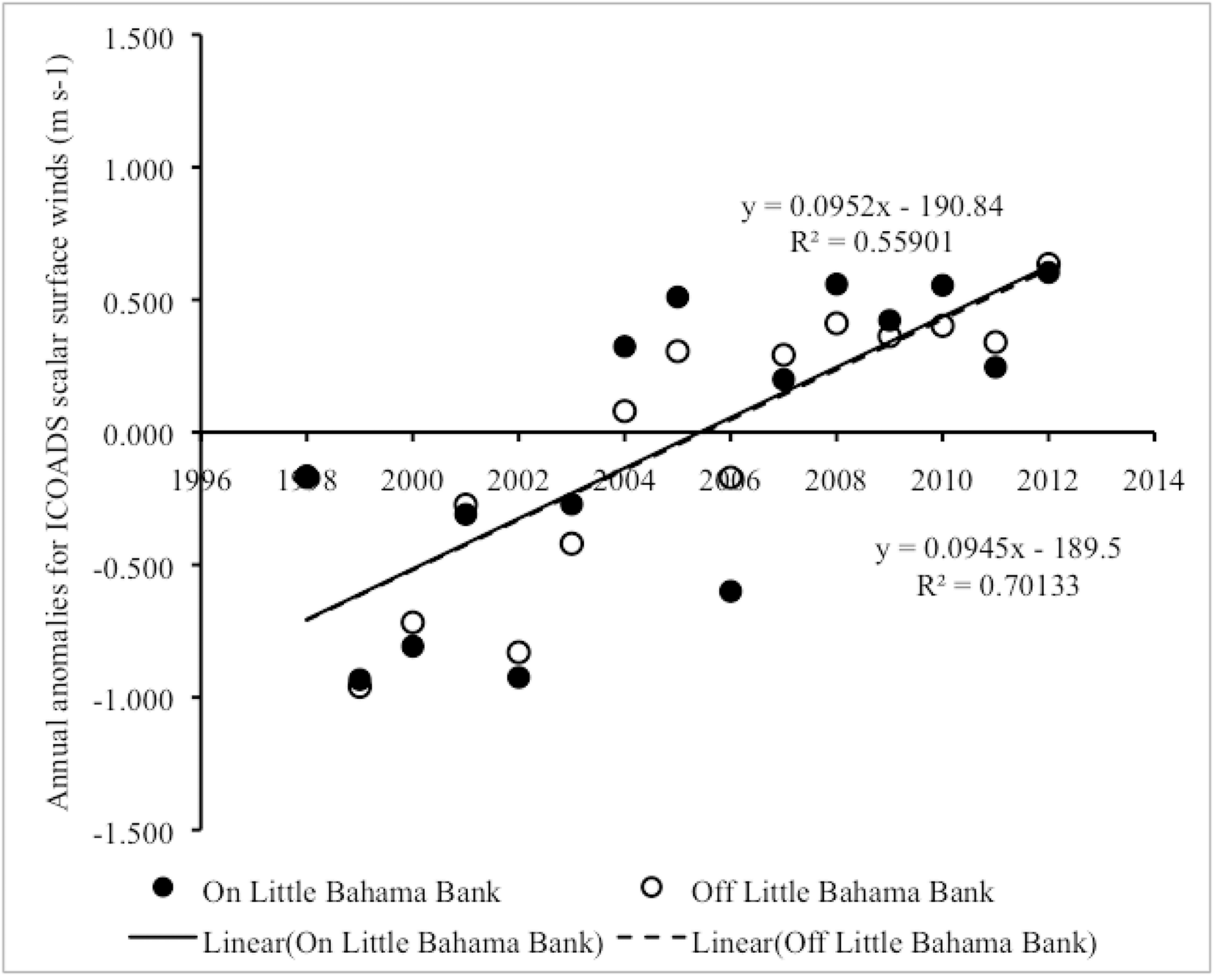
Scalar surface wind anomalies on vs. off LBB. Scatter plot of year vs. annual anomalies in surface winds on LBB vs. off LBB from 1998–2012. Trend lines, R^2^ and linear regression equations are shown for significant trends.

**Fig 12:**
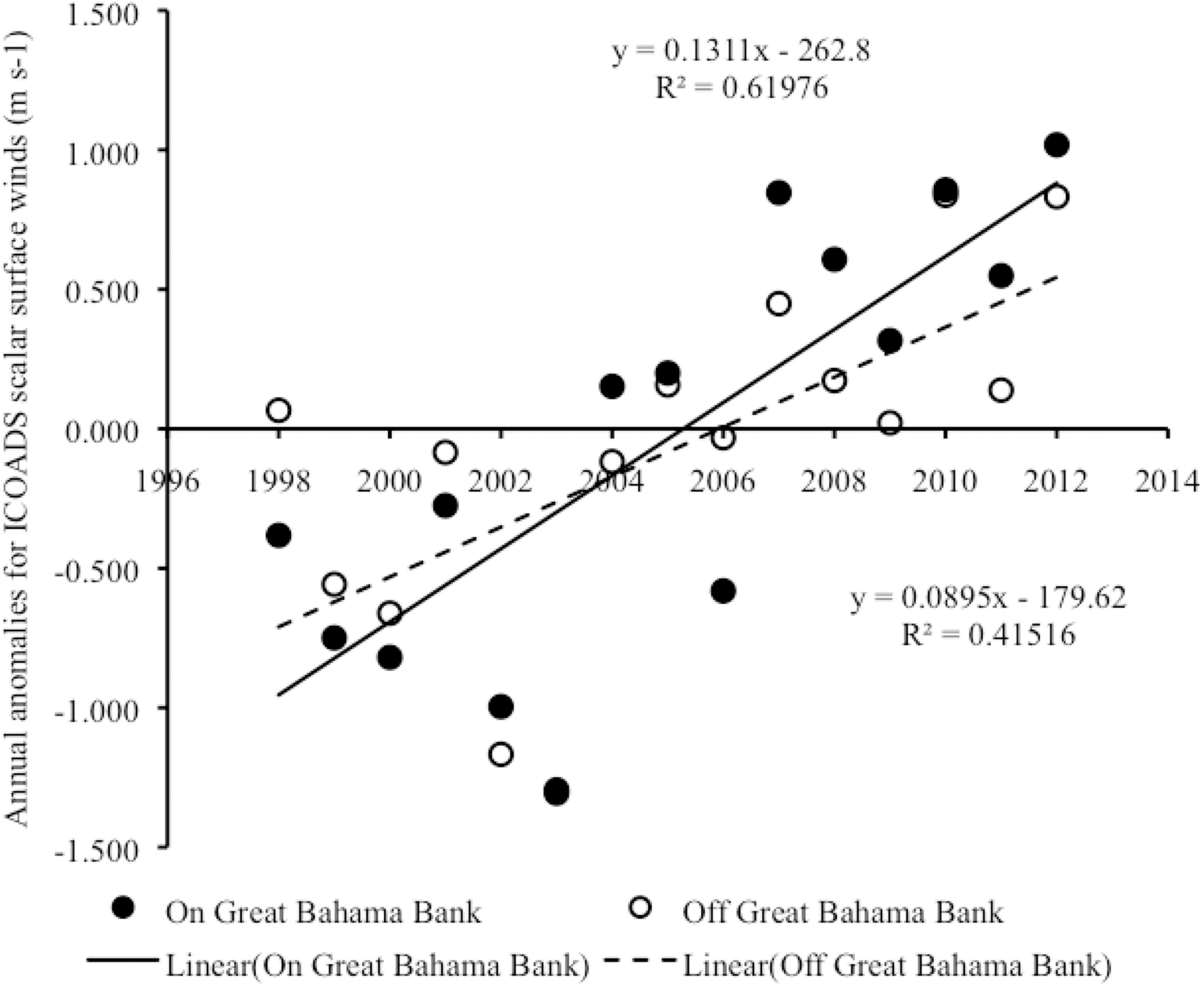
Scalar surface wind anomalies on vs. off GBB. Scatter plot of year vs. annual anomalies in surface winds on GBB vs. off GBB from 1998–2012. Trend lines, R^2^ and linear regression equations are shown for significant trends.

From 1998–2012, there was an overall positive linear correlation in year vs. annual anomalies of surface wind speed both on LBB (Pearson correlation: r_15_ = 0.79, p = 0.001) and on GBB (Pearson correlation: r_15_ = 0.76, p = 0.001) (Fig 10) (S7 Fig), with year accounting for a significant proportion of the variance in annual anomalies in surface wind speed in both areas (In LBB, R^2^ = 0.62, F_1,13_ = 20.99, p = 0.001; in GBB, R^2^ = 0.58, F_1,13_ = 17.60, p = 0.001). The microanalysis revealed that the overall increasing trend in annual anomalies in surface winds on LBB was associated with a significant positive linear correlation between annual surface wind speed anomalies and year both on LBB (Pearson correlation: r_15_ = 0.75, p = 0.001) and off LBB (Pearson correlation: r_15_ = 0.84, p < 0.001), with year accounting for a significant proportion of the variance in annual anomalies in surface winds in both areas (on LBB, R^2^ = 0.56, F_1,13_ = 16.48, p = 0.001; off LBB, R^2^ = 0.70, F_1,13_ = 30.53, p < 0.001) (Fig 11) (S8 Fig). The microanalysis also revealed that the overall increasing trend in annual anomalies in surface winds on GBB was associated with a significant positive linear correlation between annual surface wind anomalies and year both on GBB (Pearson correlation: r_15_ = 0.79, p < 0.001) and off GBB (Pearson correlation: r_15_ = 0.64, p = 0.010), with year accounting for a significant proportion of the variance in annual anomalies in surface winds in both areas (on GBB, R^2^ = 0.62, F_1,13_ = 21.19, p < 0.001; off the GBB sandbank, R^2^ = 0.42, F_1,13_ = 9.23, p = 0.010) (Fig 12) (S9 Fig).

## Discussion

Dispersal can have profound effects on the structure and stability of a population [5] and subsequent demographic and/or environmental factors can help shape the future social structure [3,14,37–43]. For 28 years the resident community of Atlantic spotted dolphins suggested a stable association scenario with long-term social clusters, high social differentiation and preferred companions [12] until sometime between fall 2012 and spring 2013 when a major distribution shift of 50% of the stable spotted dolphin community occurred, throwing both the remnant community, and the shifted community, into new situations. Responses to demographic changes may differ between populations, with varying degrees of alterations in population and/or social structure as they adapt to changing conditions [14], which can profoundly affect the survival of the individual, and structure at the community and population levels. We discuss the social and genetic implications of the unprecedented emigration event described here and the possible factors that may have contributed to the move.

### Social implications

The movement of individuals affects and is affected by the environment and other individuals [37]. An individual’s movements, through its own situation with regard to sex, age, ontogenetic status and social surroundings, may affect or be affected by others [38]. The splitting of the LBB spotted dolphin community resulted in two different communities with varying social structures that are distinctly different from their previous long-term structure. In many social species it has been shown that demographic changes can result in altered behavior and social structure [3,14,39–46]. In sperm whales it has been suggested that the stark differences in social structure between whales in the Pacific vs. Atlantic may be attributed to the anthropogenic destruction of genetic lineages [44] where the devastation of social units due to intense whaling in the Pacific may have led to larger, less matrilineal and more socially homogenous units [45]. The results of this study indicate that the movement of such a large socially cohesive cluster altered the social patterns of the remnant and shifted communities. This could have profound impacts on the future social structure and survival of the two spotted dolphin communities (and affect the sympatric bottlenose dolphin communities with whom they regularly interact) on LBB and GBB, which are likely part of a larger genetic population.

For the RLBB dolphins after the emigration event, social differentiation remained moderate/high (likely due to differences in associations between the *Northern* and *Southern* individuals), but their overall associations were random with no preferred or avoided companions, strikingly different from their nonrandom associations documented since 1991 [12,14]. Small communities of dolphins and primates have been shown to have increased cohesiveness and less fission/fusion dynamics, with more time spent with all other members of the population [40,47]. Smaller social groups may need more individuals to maintain normal social and behavioral functions, thus making recruitment of individuals easier [48] and/or necessary. This did not occur between the clusters on LBB after the hurricanes and loss of over 30% of the population [14] nor after the mass emigration event. This provides further support to the finding of [14] that the clusters are an integral part of the community structure, remaining in some capacity regardless of demographic changes. It may also take longer than three years for the individuals to adapt to the new conditions; in this study site it took more than five years for ‘normal’ aggressive behaviors during interspecies encounters to re-occur following hurricanes and social restructuring [22], thus future research may reveal nonrandom associations and a similar or different social structure. Regardless of time frame however, the changes observed will have important implications regarding survivability of individuals, social clusters/social structure, and community as a whole if outside individuals and genes are not integrated (see genetic section below).

The low social differentiation observed within each social cluster on LBB prior to the emigration event indicates that there was some complexity in the associations between individuals within in each cluster. For each cluster the levels remained relatively consistent between 2007–2009 and 2010–2012 (the drop for the *Southern* cluster was likely an artifact because fewer individuals were included in the analysis based on number of sightings). Interestingly the social differentiation for the MGBB dolphins was 0, indicating relationships between members were completely homogenous. This is a large change from the ~0.30 social differentiation of the *Central* cluster prior to the move and also indicates (along with the Mantel test showing no difference within/between clusters) that the *Northern* and *Southern* individuals became fully integrated into the new MGBB cluster. Immigration carries costs (*e*.*g*., increased aggression, decreased foraging and energetic travel costs) which can be high [4], with such a large group moving as one, there may have been more incentive to increase cohesiveness to maintain normal behaviors in an unknown situation. Although we do not have information on social differentiation or associations for RGBB dolphins prior to the event, the current ~0.29 social differentiation indicates a similar diversity of association as seen in the LBB social clusters prior to the move. Associations between MGBB and RGBB dolphins were extremely low indicating that MGBB dolphins did not integrate into the resident community, but maintained themselves as a distinctly different social cluster.

Interestingly there were three male individuals that seemed to link the very thin connection between the two clusters on GBB. There is often a sex bias in acceptance of female *vs*. male immigrants, and in many cases female immigrants may find more resistance and male immigrants find less from residents [1,3,48]. In many dolphin populations males tend to have larger home ranges [17,49–50]. Thus it may not be uncommon for residents to interact with various unknown males, which may make it easier for immigrant males, like these three, to integrate [3]. These individuals may begin to cement relationships between the immigrants and residents possibly becoming “centralized brokers”, who in some social scenarios, may play a more important role in the connectivity within a network [41]. The future structure of this community will depend greatly on the amount of interaction and ultimate integration of these two clusters with varied social structures.

In these spotted dolphins, females generally remain in their natal cluster [12], however there were matriline splits where one dolphin stayed on LBB and the other moved to GBB. Interestingly this type of split did not occur after losing over 30% of the community following the hurricanes [14]. A similar event occurred in killer whales following demographic losses related to the *Exxon Valdez* oil spill, where a subpod split from the original matrilineal pod and began consistently associating with another pod [42] and may have been due to the loss of related females tying the pod together. In both cases, these splits are unprecedented and unexplained. This could indicate that for some individuals strong social familiarity with other conspecifics (vs. matrilineal relationships) may have a stronger influence on associations and behavior. This may be more important for females than males, as social familiarity is an important component of female associations [12,51]

Male alliances were observed in both MGBB and RGBB dolphins, though with some altered patterns. The most interesting is three of the four MGBB alliances were formed when the individuals were juveniles and two of these are individuals that remained intact (although with some changes) since the hurricanes and through the move. One of the three RGBB alliances was also between speckled individuals. Alliance level associations between juvenile spotted dolphins had not been documented until after the hurricanes [14], as relationships like this usually crystallize during sexual maturity [12,52]. Factors that alter social behavior may affect young animals to a greater degree [53], and the loss of individuals after the hurricanes may have effectively sped up the development of alliance formation [14]. This idea is supported by the results presented here, and further support that environmental and demographic changes can influence current and future alliance formation.

The other notable difference is that two of the four MGBB alliances and one of the three RGBB alliances may have as many as four alliance partners. Although it is difficult to determine the exact nature of these alliances from their CoAs at this point, it is clear that there may be a more complicated structure than has previously been seen. Long-term stable dolphin male alliances are generally formed between pairs or trios of individuals [12,49,54]. Some also have second order alliances of strong associations between members of different alliances that can vary in level of stability over years [12,14,55–56] Alliance size and formation are affected by the number of competing males and the factors that impact this including density of females, operational sex ratio, and encounter rate of females [57]. It has also been shown that environmental disturbance/large demographic changes can affect alliance formation, as seen with the juvenile alliances formed after the hurricanes on LBB [14]. The combination of normal alliance formation factors and the unprecedented emigration event may have created the possibility of an alliance with four members (vs. two pair alliances with strong associations) to be viable and possibly necessary. Further research into the stability of these associations over time will provide insight into whether this is a stable scenario, or a temporary status during times of change.

Social behavior and dispersal may be connected by feedback loops so that dispersal patterns may change social behavior, which in turn may modify dispersal patterns [38]. There is behavioral flexibility between delphinid populations indicating that social variability is a common response to environmental variability [47,58], but may also be important in surviving environmental or demographic changes that can affect social structure and sociality [3,14]. This is evident in this study as well, and further supports the suggestion that responses to demographic upheaval differ between populations and/or species, with varying degrees of social structure changes as the individuals adapt to new conditions [14].

### Genetic and population implications

The change in community groups has major implications for genetic diversity. Male reproductive success and female strategies may change depending on sex ratio and/or resource availability and these factors may influence the overall mating system of the community [59].

If we consider the animals that relocated separate from those that remained on LBB, there are differing potential outcomes. For those animals that relocated to GBB, the outcome is likely positive. It is not surprising that the average relatedness among the animals that moved to GBB was similar to the *Central* cluster, because the entire *Central* cluster moved to GBB with a few additional animals. Previous research indicates that the *Central* cluster, being the largest, could function as a self-contained social and reproductive unit [15]. The number of males and females could support mating within the cluster even though mating between clusters was indicated through genetic paternity assignments. Therefore, when moving to a new location, the cluster will likely remain relatively constant. The group that dispersed may face integration issues with the existing spotted and bottlenose dolphins on GBB but we expect mating to occur between members of the RGBB group and the relocated MGBB group, even if they do not fully integrate socially. With the addition of new mating opportunities through the resident group on GBB, the gene pool will likely expand and provide increased genetic diversity over time. The same reciprocal benefit is expected for the resident animals of GBB.

On the other hand, given the reduced number of spotted dolphins in the remnant community on LBB, especially males, mating opportunities are greatly reduced. The animals that moved to GBB had a greater *r*-value than those that stayed indicating that the animals left on LBB are less closely related than those that moved. However, given the small number of genotyped animals from the *Northern* and *Southern* clusters, the data should be interpreted cautiously. The level of relatedness may be underestimated as a result of missing genotypes from close relatives to those that were genotyped. With a low level of genetic diversity and closely related individuals, the remnant animals may experience at least some level of inbreeding over time. Without an influx of new genes, this could result in decreasing levels of genetic diversity and potential mating between closely related animals. Such negative outcomes are expected among the remaining animals on LBB unless new mates become available. It remains to be seen whether new spotted dolphins will enter the area. However, the previous low immigration rates may make an influx in immigration an unlikely scenario. This is especially true if the exodus was a result of limited resources. In that case, we do not expect large numbers of new animals to immigrate into the area simply because the habitat is resource limited.

It is possible that rather than new spotted dolphins immigrating into the area, new bottlenose dolphins will enter the area. Although resource limits could still be a limiting factor for new immigration of any species, bottlenose and spotted dolphin food resources do not overlap completely [60]. Because we have observed increased immigration of bottlenose dolphins in to the LBB study area following the hurricanes [3], it is reasonable to expect bottlenose dolphins to fill the niche if resources are available. With increasing numbers of bottlenose dolphins and smaller numbers of spotted, the possibility for hybridization increases. For decades we have observed mating behaviors on LBB between spotted and bottlenose dolphins [19], often unidirectional [20]. There are at least 15 anomalous bottlenose dolphins on LBB that exhibit varying degrees of uncharacteristic ventral spotting [61]. Furthermore, a suspected bottlenose—spotted hybrid calf was observed on GBB; the suspicion of hybridization was based on intermediate morphology between the two species [61]. Although the hybrid was not genetically confirmed observations of anomalous individuals lends strong support to the idea that hybridization already occurs between these species and it is reasonable to expect hybridization given the relatively common hybridization of bottlenose dolphins with other species [62–65]. In the short term, if hybridization occurs, it opens up new mating opportunities to the remnant population of spotted dolphins and provides a mechanism to bolster their population size. Furthermore, hybridization may open the possibility of speciation mechanisms. Although in the short term it may be positive to increase hybridization, the long-term effects may be challenging. For instance, if the majority of mates are bottlenose, an imbalanced species ratio may cause mates to become a limited resource for spotted dolphins and it may be difficult for the spotted community to rebuild its numbers on LBB. In the case of an imbalanced species ratio, it is not known whether the remaining spotted dolphins will eventually relocate in order to access additional mates or if they will travel longer distances to temporarily access mates similar to long-finned pilot (*Globicephala melas*) or killer whales (*Orcinus orca*). Long-finned pilot whales and killer whales live in matrifocal groups where males may remain with their natal group and social groups, at times, aggregate and provide temporary opportunities to breed with non-related individuals [66–67]. In the event that hybridization between species increases on LBB, there are fitness risks to consider for the hybrid offspring. In many instances, hybrids are not viable although the observation of a suspected hybrid calf in good health [61] indicates this may not be a concern for *Tursiops—Stenella* hybrids. Even though a hybrid calf survives, there are higher instances of sterility, especially in the heterogametic sex [68]. Such sterility would reduce the number of breeding males on LBB even further and over time it is possible that spotted dolphins would decrease further in numbers, especially if new spotted dolphins do not immigrate to LBB.

### Habitat and survival implications

Major dramatic shifts of habitat in several cetacean species have been associated with changes in prey field [69]. For example, [70] reported a gradual departure of humpback whales (*Megaptera novaeangliae*) from Stellwagen Bank in the Southern Gulf of Maine, a major feeding ground in the mid-1970s through mid-1980s. From 1990 to 1994 the number of identified humpbacks decreased from 258 to 7 and from 1988–1994, the mean number of humpbacks identified per day dropped from 17.7 to 0.9 respectively. The major prey item of humpback whales off Stellwagen Bank in the 1970s and 1980s was sand lance (*Ammodytes spp*.). Echo-sounder surveys in 1990 and 1992 showed a dramatic decline in prey trace levels from 19.1% of the vertical water column to 2.8% respectively. Data from Jeffrey’s Ledge, 22 km north of Stellwagen Bank revealed a sudden increase in humpbacks in 1992 with 64% individuals that had previously been identified off Stellwagen Bank in the late 1980s. The population shift in humpbacks from Stellwagen Bank to Jeffery’s Ledge appeared to be associated with recovering herring populations.

The dramatic shift of over 50% of the historically stable Atlantic spotted dolphin community from LBB to GBB between fall 2012 and spring 2013 appeared to be associated with changes in several oceanographic factors that may likely have influenced availability of prey. From 1998 through 2012 (the final year in which the LBB Atlantic spotted dolphin community was still intact), LBB showed a significant cooling trend that could be traced to the LBB sandbank vs. the adjacent deep waters. Over this same period, LBB also witnessed a significant decrease in chlorophyll production, which could be traced to reductions both on and off the LBB sandbank. No significant changes in either of these factors were witnessed for the same period on GBB. The contrast between these gradual oceanographic trends on LBB from 1998–2012 and the dolphin community’s sudden dramatic shift between 2012 and 2013 suggests that the community may have been under an increasing stress that eventually reached a tipping point. Given the environmental factors involved (sea surface temperature and chlorophyll), and the current complexity of physical oceanographic features [71–73] the most likely candidate is a lack of sufficient food resources. Historically, only the *Central* cluster was documented feeding off the deep-water edge at night on flying fish and squid [13], suggesting that this offshore food source may have been compromised for this specific group of dolphins.

In terms of trends from 2013 onward, there were no significant changes in annual anomalies in either sea surface temperature or chlorophyll on either LBB or GBB although there may have been an emerging warming trend on both locations. With regard to scalar surface wind speeds, an examination of annual anomalies revealed a significant trend towards greater surface wind speeds from 1998–2012 both on LBB and GBB, which in each area was associated with increasing surface wind speeds across years both on and off the sandbanks. Surface winds, including increased speeds and changing predominate wind directions, can have an effect on nutrient replacement and production [74]. However, because greater surface wind speeds were observed on both LBB and GBB during the period leading up to the dolphin relocation event, other factors are likely to have contributed to the sea surface temperature cooling trend and collapse in chlorophyll production on LBB but not on GBB.

Because the exodus occurred between September 2012 and May 2013 we do not know if the dolphins moved gradually or as a group. The unprecedented shift in residency of LBB dolphins suggests a complex response to potential environmental changes, and may have profound effects on the social and genetic structure, including hybridization of both species, of both communities on LBB and GBB. Ecological forces, sometimes linked with changes in prey density, have been shown to act strongly on the social behavior of cetaceans [52,75–78] and the genetic composition of mammal populations [79]. Considerable behavioral plasticity in response to stressors like these has been documented in many marine mammal species, however the limits of this plasticity are unknown [78]. Because of our findings it seems likely that changing environmental variables on LBB may have led to a crash in the food chain, causing an unprecedented move of resident animals to a new location. It remains unknown whether this is a permanent exodus of the displaced dolphins, or a temporary shift in location due to resources. Oceanographic factors should be monitored in the future to assess these changing habitat features on the social and location adjustments of this dolphin community.

## Supporting information

S1 Fig Association matrix for hierarchical cluster. Analysis and nMDS for 2013 to 2015 on Great Bahama Bank (GBB).
(XLSX)

S2 Fig Supplemental file for hierarchical cluster. Analysis and nMDS for 2013 to 2015 on Great Bahama Bank (GBB).
(XLSX)

S3 Fig Scatter plot of year versus annual anomalies in sea surface temperature (°C) for the combined shallow and adjacent deep-water areas on Little Bahama Bank and Great Bahama Bank from 1998–2012. (DOCX)

S4 Fig Scatter plot of year versus annual anomalies in sea surface temperature (°C) on and off Little Bahama Bank from 1998–2012. (DOCX)

S5 Fig Scatter plot of year versus annual anomalies in surface chlorophyll A production for the combined shallow and adjacent deep-water areas on Little Bahama Bank (LBB) and on Great Bahama Bank (GBB) from 1998–2012. (DOCX)

S6 Fig Scatter plot of year versus annual anomalies in surface chlorophyll A production on and off Little Bahama Bank from 1998–2012. (DOCX)

S7 Fig Scatter plot of year versus annual anomalies in surface winds for the combined shallow and adjacent deep-water areas of Little Bahama Bank and of Great Bahama Bank from 1998–2012. (DOCX)

S8 Fig Scatter plot of year versus annual anomalies in surface winds on and off Little Bahama Bank from 1998–2012. (DOCX)

S9 Fig Scatter plot of year versus annual anomalies in surface winds on and off Great Bahama Bank from 1998–2012. (DOCX)
