# Supplementary material for: Exodus! Large-scale displacement and social adjustments of resident Atlantic spotted dolphins (Stenella frontalis) in the Bahamas
Source: PLoS One. 2017 Aug 9;12(8):e0180304. doi: 10.1371/journal.pone.0180304 (PMC5549894; doi:10.1371/journal.pone.0180304)
Supplement: S3 Fig — (DOCX) [file pone.0180304.s003.docx]

S6 Fig. Scatter plot of year versus annual anomalies in sea surface temperature (°C) for the combined shallow and adjacent deep-water areas on Little Bahama Bank and Great Bahama Bank from 1998-2012

|  | Annual Anomalies in Sea Surface Temperature (°C) | |
| --- | --- | --- |
| Year | Little Bahama Bank | Great Bahama Bank |
| 1998 | 0.3967 | 0.3427 |
| 1999 | 0.145 | 0.0969 |
| 2000 | -0.1025 | -0.1298 |
| 2001 | -0.2933 | -0.2448 |
| 2002 | 0.2642 | 0.1977 |
| 2003 | 0.2592 | 0.3194 |
| 2004 | -0.1525 | -0.2198 |
| 2005 | -0.2258 | -0.2273 |
| 2006 | -0.0992 | -0.0173 |
| 2007 | 0.0825 | 0.1827 |
| 2008 | -0.3067 | -0.1581 |
| 2009 | -0.0725 | -0.0106 |
| 2010 | -0.2608 | -0.1914 |
| 2011 | -0.1617 | -0.0706 |
| 2012 | -0.1233 | -0.0189 |
