# Supplementary material for: Exodus! Large-scale displacement and social adjustments of resident Atlantic spotted dolphins (Stenella frontalis) in the Bahamas
Source: PLoS One. 2017 Aug 9;12(8):e0180304. doi: 10.1371/journal.pone.0180304 (PMC5549894; doi:10.1371/journal.pone.0180304)
Supplement: S4 Fig — (DOCX) [file pone.0180304.s004.docx]

S7 Fig. Scatter plot of year versus annual anomalies in sea surface temperature (°C) on and off Little Bahama Bank from 1998-2012

|  | Annual Anomalies in Sea Surface Temperature (°C) | |
| --- | --- | --- |
| Year | On Little Bahama Bank | Off Little Bahama Bank |
| 1998 | 0.4762 | 0.3308 |
| 1999 | 0.132 | 0.1383 |
| 2000 | -0.1188 | -0.1392 |
| 2001 | -0.298 | -0.3292 |
| 2002 | 0.3378 | 0.1608 |
| 2003 | 0.3295 | 0.1716 |
| 2004 | -0.188 | -0.1359 |
| 2005 | -0.1863 | -0.2767 |
| 2006 | -0.1247 | -0.0875 |
| 2007 | -0.0355 | 0.2266 |
| 2008 | -0.3863 | -0.1884 |
| 2009 | -0.1297 | 0.0308 |
| 2010 | -0.3463 | -0.1 |
| 2011 | -0.2363 | -0.0659 |
| 2012 | -0.2338 | 0.025 |
