# Supplementary material for: Exodus! Large-scale displacement and social adjustments of resident Atlantic spotted dolphins (Stenella frontalis) in the Bahamas
Source: PLoS One. 2017 Aug 9;12(8):e0180304. doi: 10.1371/journal.pone.0180304 (PMC5549894; doi:10.1371/journal.pone.0180304)
Supplement: S5 Fig — (DOCX) [file pone.0180304.s005.docx]

S8 Fig. Scatter plot of year versus annual anomalies in surface chlorophyll A production for the combined shallow and adjacent deep-water areas on Little Bahama Bank (LBB) and on Great Bahama Bank (GBB) from 1998-2012

|  | Annual Chlorophyll Anomalies (log10(mg/m3)  Surface Chlorophyll A) | |
| --- | --- | --- |
| Year | Little Bahama Bank | Great Bahama Bank |
| 1998 | 0.0333 | -0.0275 |
| 1999 | 0.0525 | -0.0433 |
| 2000 | 0.0375 | -0.0133 |
| 2001 | 0.0466 | -0.0358 |
| 2002 | 0.0283 | 0.0134 |
| 2003 | -0.0059 | -0.0633 |
| 2004 | -0.0075 | 0.035 |
| 2005 | -0.0184 | -0.0308 |
| 2006 | 0.0025 | 0.0242 |
| 2007 | -0.0092 | 0.06 |
| 2008 | -0.0234 | 0.0109 |
| 2009 | 0.0291 | 0.0767 |
| 2010 | 0.005 | -0.03 |
| 2011 | -0.0534 | 0.0192 |
| 2012 | -0.0392 | -0.0091 |
