# Supplementary material for: Exodus! Large-scale displacement and social adjustments of resident Atlantic spotted dolphins (Stenella frontalis) in the Bahamas
Source: PLoS One. 2017 Aug 9;12(8):e0180304. doi: 10.1371/journal.pone.0180304 (PMC5549894; doi:10.1371/journal.pone.0180304)
Supplement: S6 Fig — (DOCX) [file pone.0180304.s006.docx]

S9 Fig. Scatter plot of year versus annual anomalies in surface chlorophyll A production on and off Little Bahama Bank from 1998-2012

|  | Annual Chlorophyll Anomalies (log10(mg/m3) Surface Chlorophyll A) | |
| --- | --- | --- |
| Year | On Little Bahama Bank | Off Little Bahama Bank |
| 1998 | 0.0398 | 0.008 |
| 1999 | 0.0765 | 0.013 |
| 2000 | 0.0606 | 0.0022 |
| 2001 | 0.069 | 0.0063 |
| 2002 | 0.0556 | -0.0145 |
| 2003 | -0.0144 | 0.0097 |
| 2004 | -0.0119 | 0.0063 |
| 2005 | -0.0269 | -0.007 |
| 2006 | 0.0031 | -0.0053 |
| 2007 | -0.0102 | -0.0103 |
| 2008 | -0.036 | -0.0062 |
| 2009 | 0.049 | 0.0055 |
| 2010 | 0.0098 | 0.0047 |
| 2011 | -0.0785 | -0.0128 |
| 2012 | -0.056 | -0.0178 |
