# Supplementary material for: Exodus! Large-scale displacement and social adjustments of resident Atlantic spotted dolphins (Stenella frontalis) in the Bahamas
Source: PLoS One. 2017 Aug 9;12(8):e0180304. doi: 10.1371/journal.pone.0180304 (PMC5549894; doi:10.1371/journal.pone.0180304)
Supplement: S7 Fig — (DOCX) [file pone.0180304.s007.docx]

S10 Fig. Scatter plot of year versus annual anomalies in surface winds for the combined shallow and adjacent deep-water areas of Little Bahama Bank and of Great Bahama Bank from 1998-2012

| Year | Annual anomalies for ICOADS scalar surface winds (m s^-1^) | |
| --- | --- | --- |
|  | Little Bahama Bank | Great Bahama Bank |
| 1998 | -0.1449 | -0.2327 |
| 1999 | -0.9282 | -0.6852 |
| 2000 | -0.7499 | -0.7669 |
| 2001 | -0.2741 | -0.211 |
| 2002 | -0.9149 | -1.0535 |
| 2003 | -0.3691 | -1.2994 |
| 2004 | 0.1768 | 0.0648 |
| 2005 | 0.4326 | 0.1856 |
| 2006 | -0.3874 | -0.3985 |
| 2007 | 0.2451 | 0.714 |
| 2008 | 0.4859 | 0.4623 |
| 2009 | 0.3851 | 0.2181 |
| 2010 | 0.4693 | 0.8498 |
| 2011 | 0.2726 | 0.4131 |
| 2012 | 0.6159 | 0.9556 |
