# Supplementary material for: Exodus! Large-scale displacement and social adjustments of resident Atlantic spotted dolphins (Stenella frontalis) in the Bahamas
Source: PLoS One. 2017 Aug 9;12(8):e0180304. doi: 10.1371/journal.pone.0180304 (PMC5549894; doi:10.1371/journal.pone.0180304)
Supplement: S8 Fig — (DOCX) [file pone.0180304.s008.docx]

S11 Fig. Scatter plot of year versus annual anomalies in surface winds on and off Little Bahama Bank from 1998-2012

| Year | Annual anomalies for ICOADS scalar surface winds (m s^-1^) | |
| --- | --- | --- |
|  | On Little Bahama Bank | Off Little Bahama Bank |
| 1998 | -0.1646 | -0.169 |
| 1999 | -0.9329 | -0.9599 |
| 2000 | -0.8071 | -0.7174 |
| 2001 | -0.3096 | -0.2732 |
| 2002 | -0.9246 | -0.8299 |
| 2003 | -0.2712 | -0.4199 |
| 2004 | 0.3238 | 0.0801 |
| 2005 | 0.5104 | 0.306 |
| 2006 | -0.5996 | -0.1707 |
| 2007 | 0.1996 | 0.2918 |
| 2008 | 0.5588 | 0.411 |
| 2009 | 0.4221 | 0.3626 |
| 2010 | 0.5546 | 0.4018 |
| 2011 | 0.2454 | 0.3401 |
| 2012 | 0.6029 | 0.6343 |
