# Supplementary material for: Exodus! Large-scale displacement and social adjustments of resident Atlantic spotted dolphins (Stenella frontalis) in the Bahamas
Source: PLoS One. 2017 Aug 9;12(8):e0180304. doi: 10.1371/journal.pone.0180304 (PMC5549894; doi:10.1371/journal.pone.0180304)
Supplement: S9 Fig — (DOCX) [file pone.0180304.s009.docx]

S12 Fig. Scatter plot of year versus annual anomalies in surface winds on and off Great Bahama Bank from 1998-2012.

| Year | Annual anomalies for ICOADS scalar surface winds (m s^-1^) | |
| --- | --- | --- |
|  | On Great Bahama Bank | Off Great Bahama Bank |
| 1998 | -0.3818 | 0.0664 |
| 1999 | -0.7493 | -0.5577 |
| 2000 | -0.8193 | -0.6611 |
| 2001 | -0.2751 | -0.0844 |
| 2002 | -0.9959 | -1.1669 |
| 2003 | -1.2943 | -1.3061 |
| 2004 | 0.1524 | -0.1177 |
| 2005 | 0.1999 | 0.1573 |
| 2006 | -0.5809 | -0.0319 |
| 2007 | 0.8466 | 0.4489 |
| 2008 | 0.6074 | 0.1731 |
| 2009 | 0.3166 | 0.0214 |
| 2010 | 0.8566 | 0.8373 |
| 2011 | 0.5491 | 0.1389 |
| 2012 | 1.0182 | 0.8323 |
